# mRNA turnover dynamics are affected by cell differentiation and loss of the cytosine methyltransferase Nsun2

**DOI:** 10.1093/nar/gkaf995

**Published:** 2025-10-16

**Authors:** Isabel Delazer, Ingo Bauer, Teresa Rummel, Kamila Nykiel, Dietmar Rieder, Magdalena Fickl, Valentin Tumler, Anna Razkova, Matthias R Schaefer, Thalissa Scheed, Matthias D Erlacher, Florian Erhard, Ronald Micura, Alexandra Lusser

**Affiliations:** Institute of Molecular Biology, Biocenter, Medical University of Innsbruck, 6020 Innsbruck, Austria; Institute of Molecular Biology, Biocenter, Medical University of Innsbruck, 6020 Innsbruck, Austria; Institute for Virology and Immunobiology, University of Würzburg, 97078 Würzburg, Germany; Faculty for Informatics and Data Science, University of Regensburg, 93053 Regensburg, Germany; Institute of Molecular Biology, Biocenter, Medical University of Innsbruck, 6020 Innsbruck, Austria; Institute of Bioinformatics, Biocenter, Medical University of Innsbruck, 6020 Innsbruck, Austria; Institute of Molecular Biology, Biocenter, Medical University of Innsbruck, 6020 Innsbruck, Austria; Institute of Molecular Biology, Biocenter, Medical University of Innsbruck, 6020 Innsbruck, Austria; Institute of Genomics and RNomics, Biocenter, Medical University of Innsbruck, 6020 Innsbruck, Austria; Institute of Organic Chemistry, Center for Molecular Biosciences Innsbruck (CMBI), University of Innsbruck, 6020 Innsbruck, Austria; Division of Cell and Developmental Biology, Center for Anatomy and Cell Biology, Medical University of Vienna, 1090 Vienna, Austria; Institute of Molecular Biology, Biocenter, Medical University of Innsbruck, 6020 Innsbruck, Austria; Institute of Genomics and RNomics, Biocenter, Medical University of Innsbruck, 6020 Innsbruck, Austria; Institute for Virology and Immunobiology, University of Würzburg, 97078 Würzburg, Germany; Faculty for Informatics and Data Science, University of Regensburg, 93053 Regensburg, Germany; Institute of Organic Chemistry, Center for Molecular Biosciences Innsbruck (CMBI), University of Innsbruck, 6020 Innsbruck, Austria; Institute of Molecular Biology, Biocenter, Medical University of Innsbruck, 6020 Innsbruck, Austria

## Abstract

Nsun2 catalyzes 5-methylcytosine (m^5^C) formation in several types of RNA, including messenger RNAs (mRNAs), transfer RNAs, and other non-coding RNAs. In mRNA, m^5^C was reported to influence transcript stability. However, it is unclear if it has stabilizing or destabilizing effects. To address the role of Nsun2 in mRNA stability, we characterized the landscape of mRNA turnover dynamics during embryonic stem cell (ESC) differentiation in wild-type and *Nsun2*-mutant cells. By using an RNA labeling approach combined with thiouridine-to-cytidine-sequencing (TUC-seq), we demonstrate that mRNA synthesis and stability undergo extensive changes during normal cellular differentiation. Remarkably, a large proportion of these changes did not result in altered mRNA abundance, providing evidence for robust transcript buffering during ESC differentiation. Importantly, also the loss of Nsun2 affected mRNA turnover dynamics but not the steady-state levels of transcripts. Furthermore, our data indicate that the effect of Nsun2 on mRNA turnover was not mediated by m^5^C deposition in mRNA, which is corroborated by catalysis-independent effects of Nsun2 on translation in early ESC differentiation. In conclusion, this study demonstrates that differentiation as well as loss of Nsun2 can induce changes in mRNA turnover dynamics that are independent of mRNA methylation but consistent with a buffering mechanism to maintain constant RNA levels.

## Graphical Abstract

**Graphical Abstract:**
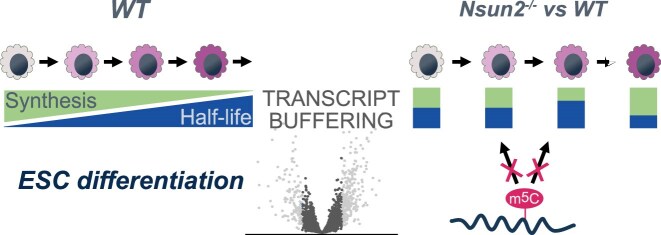

## Introduction

The NOL1/NOP2/Sun domain family member Nsun2 catalyzes the methylation of cytosines (m^5^C) in transfer RNAs (tRNAs), non-coding RNAs (ncRNAs), and messenger RNAs (mRNAs) in a structure- and sequence (5′m^5^CNGGG)-specific manner [1–9]. Strong links have been established between NSUN2 and cancer. For instance, Nsun2 was observed to be upregulated in response to the proto-oncogene Myc [10] in mice, it was implicated in cell cycle regulation [11, 12], and overexpression of human NSUN2 has been observed in numerous tumors [10, 13–15]. Various mutations in the *NSUN2* gene have been associated with neurodevelopmental defects in humans, such as growth and language retardation, facial abnormalities, reduced brain size, and autosomal recessive intellectual disability [16–18]. Studies with *Nsun2^−/−^* mice suggested that the observed phenotypes may be linked to a defect of intermediate neural progenitor differentiation into upper layer neurons and impaired cell migration and differentiation of neuroepithelial stem cells [19].

At the molecular level, the enzymatic functions of Nsun2 are best understood with regard to its role in methylating specific cytosines in the variable loop of various tRNAs [20, 21]. Loss of m^5^C at these positions was shown to increase tRNA fragmentation by the nuclease angiogenin. The resulting 5′tRNA fragments were postulated to inhibit general translation while allowing synthesis of stress-related proteins [20–22]. Methylation of cytosines in mRNA was proposed to influence both transcript stability [23–26] and protein translation [27–33]. Nevertheless, the precise function of Nsun2 in these processes remains uncertain, as the results of different studies have yielded varying outcomes. For instance, Nsun2-mediated m^5^C in mRNA was reported to promote translation by some studies [28–30, 32], whereas other studies observed a repressive effect [25, 31, 33]. Notably, mRNA stability was found to correlate positively as well as negatively with m^5^C modification [23–25]. For instance, in zebrafish embryos, the reader protein Ybx1 was shown to preferentially bind to m^5^C-containing transcripts, resulting in the recruitment of Pabp1a and increased transcript stability [23]. Likewise, human YBX1 was found to interact with ELAVL1 to increase the stability of m^5^C-modified transcripts in the context of bladder cancer [24]. In contrast, a recent study correlated the mRNA decay-promoting activity of UPF1 to the presence of m^5^C sites in target mRNAs [26].

Thus, it is currently unclear whether Nsun2 affects mRNA stability in a positive, negative, or context-dependent manner, or whether its impact on mRNA is directly mediated by mRNA methylation or indirectly, for instance, through its robust tRNA methylation activity. To address these open questions, we performed a comprehensive analysis of mRNA turnover dynamics, specifically by measuring mRNA synthesis and stability, both in the presence and absence of Nsun2. To do so, we employed a minimally invasive method based on metabolic labeling of nascent transcripts with 4-thiouridine (4sU) in conjunction with thiouridine-to-cytidine sequencing (TUC-seq) [34]. Given that mutations in human NSUN2 give rise to neurodevelopmental effects, we focused on the effects of Nsun2 ablation during differentiation of mouse embryonic stem cells (mESCs) into the neuroectodermal lineage (NEC). Our results demonstrate that loss of Nsun2 profoundly affected mRNA synthesis and stability in differentiating cells but not in self-renewing mESCs. Importantly, the data also indicate that the impact of Nsun2 function on mRNA turnover is likely independent of its ability to methylate cytosines in mRNA. Furthermore, characterization of RNA turnover dynamics in wild-type (WT) mESCs revealed extensive changes during differentiation. Remarkably, these changes were far more pronounced than the differentiation-associated alterations of steady-state transcript levels, suggesting extensive compensation between mRNA synthesis and decay in regulating mRNA levels during differentiation.

## Materials and methods

### Cell culture

mESCs (KH2) were cultured in ESC LIF + 2i media [Dulbecco’s modified Eagle’s medium (DMEM) high glucose with GlutaMAX (Gibco), 15% ESC fetal bovine serum (Gibco), 1× non-essential amino acid mix (Gibco), 1 mM sodium pyruvate (Gibco), 0.05 mM β-mercaptoethanol (Gibco), 10 μg/ml LIF (Sigma), 3 μM CHIR99021, 1 μM PD0325901 (Axon Medchem)]. The cells were cultured in gelatin-coated tissue culture dishes at 37°C and 5% CO_2_. HEK293T cells (ATCC, CRL-3216) were cultured in DMEM high glucose with GlutaMAX (Gibco), 10% heat-inactivated FBS (Gibco), and 100 U/ml penicillin/streptomycin (Gibco) at 37°C and 5% CO_2_.

### Differentiation of ESCs

mESCs were differentiated into the neuroectoderm lineage (NEC) using serum-free N2B27 medium as described previously [35]. The medium was composed of a 1:2 mixture of DMEM/F12 (Gibco) containing 1× N-2 supplement (Gibco) and Neuroectoderm medium (Gibco) containing 0.5× B-27 Supplement (with or without vitamin A; Gibco), 1× non-essential amino acid mix (Gibco), 1× GlutaMAX (Gibco), and 0.004 mM β-mercaptoethanol (Gibco). 1.5×10^4^ cells per cm^2^ were seeded in N2B27 medium (B-27 supplement without vitamin A) containing 10 μg/ml LIF. After 24 h, the medium was changed to N2B27 medium (B-27 supplement without vitamin A) containing 0.4 μM PD0325901 (Axon Medchem) but no LIF. After an additional 24 h, cells were cultured in N2B27 medium (B-27 supplement with vitamin A) containing 0.4 μM PD0325901 for two days, followed by a change of medium [N2B27; B27 supplement plus vitamin A and 1 μM LDN193189 (MedChemExpress)] and incubation for two more days.

mESCs were differentiated into embryoid bodies (EBs) using AggreWell™400 24-well plates (STEMMCELL technologies) following the manufacturer’s instructions. Briefly, to obtain EBs with 2000 cells, 2.4×10^6^ cells were seeded into each well of the AggreWell™ plate in ESC medium lacking LIF and 2i. After 48 h, the EBs were transferred onto a gelatin-coated six-well plate and allowed to grow for additional 48 h.

### Catalytic inactivation of Nsun2 in ESCs

To inactivate *Nsun2* in mouse ESCs by CRISPR/Cas9, a double-stranded DNA oligonucleotide comprising the sgRNA sequence (Supplementary Table S1) targeting the active site (T_320_C_321_ motif) [36] of Nsun2 was cloned into the *Bbs*I-digested pX458 vector (Addgene plasmid ID: 48138) [37]. The plasmid was introduced into ESCs using Lipofectamine 2000 (Invitrogen) following the manufacturer’s instructions. About 24 h after transfection, ESCs were sorted for GFP-positive cells using fluorescence-activated cell sorting (FACS). The single cells were cultured individually in 96-well plates and eventually screened for mutations in the *Nsun2* gene that resulted in the loss of the TC motif. To this end, DNA was isolated from single clones by lysing the cells in 10 mM Tris–HCl, pH 8.0, 1 mM ethylenediaminetetraacetic acid (EDTA), 30 mM KCl, 0.3% Tween, 0.3% Igepal and Proteinase K (0.1 mg/ml) for 2 h at 55°C, followed by Proteinase K inactivation at 95°C for 10 min. *Nsun2* was amplified by *Taq* DNA Polymerase (NEB) with *Nsun2* gene specific primers (Supplementary Table S1) and 1 μl lysate as template. Polymerase chain reaction (PCR) products were subcloned and sequenced.

### Generation of *Nsun2* rescue constructs and lentiviral transduction

The *Nsun2* coding sequence was amplified by PCR and inserted into *Bam*HI-linearized lentiviral transfer vector prtTA-N144 (Addgene plasmid #66810; [38] by NEBuilder cloning (New England Biolabs), replacing the rTetR gene to generate an *Nsun2* (N2_wt_) rescue construct. For a catalytic mutant (N2_cat_), pN144-N2_wt_ was subjected to site-directed mutagenesis using inverse PCR to exchange cysteine 321 to a glycine (C321G; pN144-N2_cat_). See Supplementary Table S1 for primer sequences. Virus particles were produced by transfection of HEK293T cells with a mixture containing 10 μg pN144-N2_wt_ or pN144-N2_cat_, 6.5 μg psPAX2 (Addgene #12260), and 3.5 μg pMD2.G (Addgene #12259), 0.125 mM CaCl_2_, 137 mM NaCl, 27.25 mM HEPES pH 7.0, and 0.74 mM Na_2_HPO_4_. After incubation for 24 h, the medium was changed, and cells were incubated for 48 h at 32°C. Virus particles in the supernatant were concentrated by precipitation with 8.5% PEG-6000/0.4 M NaCl overnight at 4°C and centrifugation at 7000 ×*g* for 30 min at 4°C. The resuspended virus pellet was used to transduce *Nsun2^−/^*^*−*^ ESCs. After 24 h, the medium was changed and after incubation for another 24 h, 0.3 mg/ml hygromycin was added to select for cells with stable integrations.

### Cell viability assay

ESCs were seeded in triplicates into 96-well plates at a density of 1× 10^3^ cells per well. Cell viability was measured every 24 h using the CellTiter-Glo 2.0 Assay Kit (Promega), according to the manufacturer’s instructions.

### FACS analysis

WT and *Nsun2^−/^*^*−*^ ESCs or d4 NEC-differentiated cells were fixed in ice-cold 70% ethanol and stored at −20°C overnight. Ethanol was removed by centrifugation for 5 min at 300 ×*g*, 4°C. The cell pellet was washed with PBS and subsequently resuspended in 300 μl of propidium iodide mix (0.1 mg/ml RNase A, 40 μg/ml propidium iodide in PBS). After incubation at 37°C for 10 min, cells were passed through a 200 μm filter (pluriSelect) and measured in an Attune NxT flow cytometer (Thermo Fisher Scientific) with Attune Cytometric Software (v5.3.0). The gaiting parameters were set to FSC = 130 V, SSC = 270 V, BL2 = 200 V. The cell cycle profile was monitored until reaching 30 000 events on the BL2-A/Count plot. The data were analyzed using the free online software Floreada.io (https://floreada.io) and Prism 10 (Graphpad).

### Reverse transcription qPCR

Quantitative PCR (qPCR) was carried out with WT and *Nsun2^−/−^* ESCs (Supplementary Fig. S3B) or with HEK293T cells co-transfected with methylated or unmethylated *Cap-eGFP-Apba3-poly(A)* and *Cap-fLuc-poly(A)* mRNA (Fig. 3F). RNA isolation was performed using either TRI Reagent (Sigma–Aldrich) according to the manufacturer’s suggestions or the innuPREP RNA Mini Kit 2.0 (IST Innuscreen GmbH). The RNA was treated with DNase I (1 U/μg RNA; NEB) for 30 min at 37°C and purified by acid phenol/chloroform (Roth) extraction and ethanol precipitation. Total RNA was reverse transcribed with the GoScript Reverse Transcription System (Promega) in combination with random primers (Promega) according to the manufacturer’s instructions. qPCR reactions (20 μl) were performed in technical triplicates 2× Luna Universal qPCR Master Mix (NEB), 0.5 mM gene-specific primers (Supplementary Table S1), and 40 ng complementary DNA (cDNA) in a QuantStudio 3 Real-Time PCR system (Thermo Fisher Scientific) with 40 cycles á 95°C 15 s, 60°C 1 min. mRNA expression levels were quantified with the QuantStudio 3 software for SYBR green experiments using the 2^−ΔΔCt^ method. Mouse TATA binding protein and human *GAPDH* were used as internal reference genes for Supplementary Figs S3B and S7D and Fig. 3G, respectively.

### Western blot

ESCs were lysed in RIPA buffer (25 mM Tris–HCl, pH 7.5, 150 mM NaCl, 1% Igepal, 1% SDS, 1% deoxycholate) supplemented with 1× protease inhibitor cocktail (Roche) and Super Nuclease (5 U/ml; SinoBiological). The extracts were centrifuged for 10 min at 14 000 ×*g* and 4°C. Subsequently, protein concentration was measured using Bradford's reagent (Bio-Rad), and equal amounts were mixed with Laemmli sample buffer (final concentration: 62.5 mM Tris pH 6.8, 2% SDS, 10% glycerol, 5% β-mercaptoethanol) and loaded onto a 10% sodium dodecyl sulfate–polyacrylamide gel electrophoresis (SDS–PAGE) after denaturation at 95°C for 5 min. The proteins were blotted to nitrocellulose membrane (BioRad) using the semi-wet blotting system from BioRad. The membranes were blocked in 5% milk in phosphate buffered saline containing 0.1% Tween (PBST) and incubated overnight at 4°C with the primary antibodies (rabbit α-Nsun2, 1:1000, Proteintech; mouse α-tubulin, 1:10 000, Sigma) followed by washing in PBST and incubation with HRP-coupled secondary antibodies (1:10 000, Sigma). After washing the membranes in PBST, the signal was developed by incubation with ECL (Abcam) and detected with a Fusion-SL 3500-WL system (Vilber Lourmat).

### TUC-seq

At the designated time points of differentiation (Supplementary Fig. S1), cells were labeled with 0.1 mM 4sU (dissolved in DMSO; Jena Bioscience) in cell culture media for 1 h [34, 39]. As a control, one sample received the same amount of DMSO. Subsequently, the cells were harvested, and RNA isolation was performed using the innuPREP RNA Mini Kit 2.0 (IST Innuscreen GmbH). The RNA was treated with DNase I (1 U/μg RNA; NEB) for 30 min at 37°C and purified by acid phenol/chloroform (Roth) extraction and ethanol precipitation. Two micrograms of RNA were used for treatment with TUC-seq chemistry as described [34, 39]. Briefly, the RNA was incubated at 40°C for 1 h with 0.5 mM OsO_4_ and 180 mM M NH_4_Cl in a volume of 20 μl. The RNA was purified from the conversion reaction via precipitation with 0.3 M NaOAc, pH 5.2, 2.5 volumes 100% EtOH and 0.5 μl glycogen (20 mg/ml; RNA grade, Roche). RNA was dissolved in RNase-free water, and quantity was determined by absorbance measurement at 260 nm using a NanoPhotometer (Implen). Sequencing libraries were prepared from 100 ng TUC-treated total RNA with the Zymo-Seq 3′ mRNA library Kit (Zymo Research); 12 PCR cycles were used for the final index amplification step. The samples were sequenced on an Illumina HiSeq 1500 instrument using HiSeq PE Cluster Kit v4 cBot as clustering reagent and HiSeq SBS Kit v4 as cartridge reagent (Illumina) with 150 nt paired-end reads.

### Bisulfite sequencing

Total RNA was isolated from cells using TRI Reagent (Sigma–Aldrich) followed by DNase I (1 U/μg RNA; NEB) digestion and purification by PCI as described above. Poly(A) RNA was isolated from 100 μg total RNA by two rounds of enrichment with Dynabeads^TM^ Oligo(dT)_25_ (Invitrogen) according the manufacturer’s instructions. mRNA quality was evaluated using Agilent 2100 Bioanalyzer. Bisulfite treatment was performed as described previously [40, 41] using the EZ RNA methylation Kit (Zymo Research) with minor modifications. Briefly, 1 μg poly(A) RNA was supplemented with 1 μl 1:10 diluted non-methylated ERCC RNA mix 1 (Thermo Fisher) and then subjected to 3 cycles of 10 min at 74°C and 45 min at 64°C. Desulfonation was performed on the column at 37°C for 25 min followed by RNA clean-up according to the manufacturer’s instructions and quantification by Qubit (Invitrogen) measurement. Prior to library preparation, the conversion efficiency was evaluated for selected targets by reverse transcription PCR using the GoScript Reverse Transcriptase System (Promega) in combination with random primers (Promega), and the targets were amplified with the EpiMark Polymerase (NEB) using primers corresponding to the respective deaminated sequences (i.e. C→T conversion) (Supplementary Table S1). PCR products were subcloned into pGEM-T (Promega) and multiple clones were subjected to Sanger sequencing. For next generation sequencing of the BS-treated samples, library preparation and sequencing was performed by Zymo Research using the Zymo-seq RiboFree Total RNA Library Prep Kit and an Illumina NovaSeq instrument at ∼100 Mio 150 nt paired-end reads.

### Northern blot analysis

Total RNA (10 μg) was separated on a 12% TBE (90 mM Tris, 90 mM boric acid, 2 mM EDTA)-urea polyacrylamide gel (acrylamide:bisacrylamide 29:1; 7 M urea). The gel was stained with ethidium bromide followed by RNA transfer to a Hybond™-N Membrane (Cytiva) using a Trans-Blot SD Transfer Cell (Bio-Rad). The RNA was UV crosslinked to the membrane, pre-hybridized in a solution containing 178 mM Na_2_HPO_4_, 822 mM NaH_2_PO_4_, 7% SDS for 30 min, and subsequently 12 pmol 5′-^32^P-labeled oligonucleotides (Eurofins Genomics, Supplementary Table S1) were added for overnight incubation. The membrane was washed with 2× SSC (300 mM NaCl, 30 mM sodium citrate) and 0.1% SDS, followed by 0.1× SSC and 0.1% SDS. A phosphor screen was exposed to the membrane and signals were detected on a Typhoon FLA 9500 instrument (Cytiva).

### Reporter analysis of m^5^C effect on translation and mRNA stability

#### Generation of reporter RNA

To assess the effect of an m^5^C located in the 3′UTR of the *Apba3* mRNA on translation and mRNA stability, a reporter assay in HEK293T cells was used. To this end, a plasmid was generated containing the coding region of enhanced green fluorescent protein (eGFP) fused to the 3′UTR of *Apba3* (NM_001429267.1). The insert was PCR-amplified with forward primer T7-eGFP_fw containing a T7 promoter sequence and reverse primer Apba3_rev annealing upstream of the m^5^C site in *Apba3* (see Supplementary Table S1 for sequences). In parallel, an in-house plasmid was used to generate a PCR fragment containing a 5′ T7 promoter sequence followed by full-length firefly luciferase (*fFLUC*) coding region. The *eGFP-Apba3* PCR product was used for *in vitro* transcription with the HiScribe T7 mRNA Kit with CleanCap Reagent AG (NEB) to produce capped mRNA, while the *fFLUC* product was *in vitro* transcribed using HiScribe T7 ARCA mRNA Kit with tailing (NEB) to produce capped and polyadenylated mRNA. An RNA oligonucleotide (*Apba3-m^5^C-poly(A)*) spanning nt 1956–1992 of *Apba3* mRNA with or without methylation at position C1989 (Supplementary Table S1) was obtained from Dharmacon and subjected to polyadenylation with A-Plus Poly(A) Polymerase Tailing Kit (CellScript). Capped *eGFP-Apba3* RNA and *Apba3-m^5^C-poly(A)* were fused by splint ligation using a reverse complementary DNA splinter oligo (Supplementary Table S1) as described before [42, 43] to generate *Cap-eGFP-Apba3-poly(A)* reporter mRNA. Briefly, equimolar amounts (60 pmol) of each component were mixed and denatured at 90°C, 75°C and 55°C for 3 min each followed by incubation at 22°C for 20 min. The reaction was supplemented with 2.4 μl 10× ligation buffer (NEB), 2.4 μl PEG 4000 (Fermentas), 10 U RNase Inhibitor (NEB) and 5 U of T4 RNA Ligase 2 (5 U/μl; NEB) in a total volume of 24 μl and incubated for 3.5 h at 35°C. After digestion with 2 U of DNaseI for 30 min at 37°C, the ligation products were purified by lithium chloride precipitation followed by purification with the magnetic mRNA Isolation Kit (NEB). mRNA purity and integrity were assessed with a Nanodrop spectrophotometer and by denaturing polyacrylamide gel electrophoresis (7 M urea, 4% PAA 29:1).

#### Transfection, translation, and mRNA stability test

HEK293T cells were grown to 40% confluency, followed by transfection with 500 ng *Cap-eGFP-Apba3-poly(A)* mRNA using metafectene (Biontex) as described by the manufacturer. For mRNA stability assays, a co-transfection was performed with 500 ng *Cap-fLUC-poly(A)* mRNA. GFP production was assessed at 24 h post transfection by harvesting cells and resuspending the pellet in PBS. Aliquots of 100 μl cell suspension were transferred to a black clear-bottom 96-well plate (Greiner), and fluorescence measurements were performed in quadruplicate in a plate reader (BMG LABTECH CLARIOstear Plus) instrument. For mRNA stability analysis, the pellet from double-transfected cells was extracted with TRI reagent (Sigma–Aldrich) according to the manufacturer’s recommendations, followed by Monarch RNA Cleanup (NEB). cDNA synthesis and qPCR analysis were carried out as described above with primers shown in Supplementary Table S1. *Cap-eGFP-Apba3-poly(A)* RNA levels were calculated relative to *Cap-fLUC-poly(A)* levels and *GAPDH* using the QuantStudio 3 software (2^−ΔΔCt^ method).

### Polysome profiling of ESCs

ESC and differentiated cells were lysed in 10 mM HEPES-KOH, pH 7.4, 100 mM KCl, 5 mM MgCl_2_, 0.5% Igepal, 0.1 mg/ml cycloheximide (Sigma), and the lysate was passed through a 26-gauge needle several times. After centrifuging the lysates twice for 5 min at 13 000 × *g* and 4°C, the supernatant was loaded onto a 12 ml sucrose gradient (15%–45% sucrose). The gradient was prepared with a Seton Gradient maker using 6 ml of each sucrose solution (10 mM HEPES-KOH, pH 7.5, 100 mM KCl, 5 mM MgCl_2_, and 15% or 45% sucrose). After centrifugation using swing-out rotor SW41 in an Optima XE-90 Ultracentrifuge (Beckman Coulter) at 210 100 × *g* for 2 h, the gradient was fractionated using a peristaltic pump (Cytiva) with concomitant absorbance monitoring at 260 nm in an Äkta Pure instrument (Cytiva).

### *In vivo* labeling of proteins with ^35^S-methionine

1.5 × 10^4^ cells per cm^2^ were seeded in 35 mm TC dishes in ESC media, and differentiation into NEC was carried out as described above. At d0 (= ESCs), d1, d4, and d6 of NEC differentiation, the cells were starved for 30 min in DMEM medium lacking cysteine and methionine. Subsequently, half of the starvation media was replaced by labeling media containing 0.05 mCi/ml ^35^S-methionine in the respective differentiation medium. After 30 min the cells were washed twice in 1× PBS and then lysed in RIPA buffer as described above. Equal amounts of protein extracts were subjected to 10% SDS–PAGE; gels were stained with Coomassie Brilliant Blue, dried on Whatman paper for 45 min at 70°C in a slab gel dryer (Cleaver Scientific), and finally exposed to a phosphor screen overnight. Signals were detected on a Typhoon FLA 9500 instrument (Cytiva).

### Data analysis

#### TUC-seq

TUC-seq data were processed through the GRAND-SLAM pipeline [44]. Sequencing adapters were initially trimmed using SeqPurge (version 2022_10) [45]. Subsequently, another trimming step was performed with cutadapt (version 3.5) using the adapter sequence “-a AAAAAAAAAAAAAAAA” [46]. Unique molecular identifiers (UMIs, 8 nt) were moved to the header of R2, and R1 was subsequently discarded. This was done using FastqFilter from the GEDI toolkit (version 1.0.5) with the parameters “-overwrite -umi 8,6,0,0” [47]. Then, bowtie2 (version 2.3.0) was applied to align reads against the rRNA (NR_046233.2) and *Mycoplasma* databases, using the default parameters [48]. The remaining reads were aligned to the mouse genome (GRCm38) using STAR (version 2.7.10b) with parameters “- -outFilterMismatchNmax 40 - -outFilterScoreMinOverLread 0.3 - -outFilterMatchNminOverLread 0.3 - -alignEndsType Extend5pOfReads12 - -outSAMattributes nM MD NH” [49]. Mismatches with a PHRED score below 28 were filtered from the alignment files using GEDI BamPhredFilter [47]. Bam files were then converted into CIT files using GEDI Bam2CIT, and mapped reads were deduplicated with GEDI DedupUMI with the parameter “-mm” [47]. CIT files were then merged utilizing GEDI MergeCIT, followed by processing with GRAND-SLAM (version 2.0.7b) using the parameters “-trim5p 40 -modelall” to generate read counts and NTR values at the gene level, considering all reads that align with at least one isoform of a gene [44, 47]. Because it has been noted that 4sU labeling can lead to underrepresentation of short-lived transcripts (“drop-out”) among mapped reads due to technical reasons [50], 4sU drop-out correction was performed according to [51]. Downstream analyses including correction for 4sU dropout were conducted in R (version 4.3.1) applying the package grandR (version 0.2.5) [52]. GC content and 3′UTR length were determined for mouse canonical transcripts using R-packages biomaRt (version 2.65.0) and AnnotationHub (version 3.99.6). Data visualization and export were performed using R-packages circlize (version 0.4.16), dplyr (version 1.1.4), eulerr (version 7.0.2), ggplot2 (version 3.5.1), grandR (version 0.2.5), openxlsx (version 4.2.5.2), purr (version 1.0.2), reshape2 (version 1.4.4), and scales (version 1.3.0).

#### RNA expression analysis

Differential gene expression and corresponding Wald statistics were determined with the grandR and DESeq2 packages using the grandR LFC and PairwiseDESeq2 wrapper functions, respectively [52, 53].

#### Bisulfite sequencing

Raw sequencing data were first sub-sampled to achieve an equal number of 106 million reads per sample. Sequencing adapters were removed from raw BS-seq reads using Trimgalore before the reads were aligned to the *Mus musculus* genome (GRCm39, GENCODE release M27) extended with the ERCC sequences using meRanGs from the meRanTK software collection (v1.3.0) [54]. meRanCall was used for m^5^C candidate detection with following parameter settings: allowed duplicates < 20 (-md 20), coverage > 20 reads (-mcov 20), C-cutoff of 3 (-C_cutoff 3), signal-to-noise rate threshold 0.9 (-snr 0.9), and minimum methylation rate > 0.1 (-mr 0.1). The gene-specific conversion rate (-grc) was used to determine the statistical significance (*P*-value) of the m^5^C candidates employing a Fisher’s exact test. For m^5^C candidates outside gene regions as defined from Ensembl GTF annotation files, a conservative conversion rate of 0.9 was used for significance calculation. Inspection of the M-bias plots revealed that some samples exhibited insufficient bisulfite conversion. Consequently, these samples were omitted from subsequent analyses. As a result, our final dataset comprised two replicates for each genotype and time point, with the exception of day 6 (d6), for which only one replicate met our quality criteria. To identify robust candidate m^5^C sites, we developed a custom R script to generate a consensus list of sites present in all replicates of a given condition. The script incorporated Benjamini-Hochberg correction for multiple hypothesis testing, and only sites with a false discovery rate (FDR) below 0.05 were retained for subsequent analysis [41]. Final candidate sites were annotated by ChIPseeker (v1.34.1) [55, 56].

#### Other bioinformatic and statistical analyses

To plot heatmaps, normalized gene expression or half-life (HL), data of replicates were summarized, transformed to z-scores, and k-means clustered with eight centers using the grandR (version 0.2.5) and ComplexHeatmap (version 2.21.0) R packages [52, 57]. For the heatmap shown in Supplementary Fig. S7B, normalized expression data of genes showing significant expression changes during differentiation (|log_2_fc| > 1, *P*< .05) were transformed to z-scores and k-means clustered with four centers. Venn analyses were performed using the systemPipeR (version 2.11.6) package in R [58]. Over-representation analysis (geneontology and pathway) was performed with WebGestalt (https://2019.webgestalt.org/) entering the Ensembl geneIDs [59]. The mouse genome was used as background list. Differences in HL and synthesis rates (S) were assessed by bootstrap sampling (20 000 iterations) using package boot (version 1.3–31) in R. Statistical significance was determined when the 95% confidence interval (CI) excluded zero. For other statistical analyses, Prism 10 (Graphpad) was used.

### Artificial intelligence

DeepL Write and GPT 40 were used for English language editing.

## Results

### Changes in steady-state levels and turnover rates of mRNA during neuroectoderm differentiation

In order to elucidate the potential effects of Nsun2 on mRNA stability in the context of mESCs and their differentiation into the neuroectodermal lineage, we first sought to gain a general view of the differentiation-associated changes in mRNA levels and turnover rate in WT mESCs. We employed TUC-seq [34] to measure RNA synthesis, HL, and total abundance in ESCs compared to different time points of NEC differentiation. To this end, nascent RNA was pulse-labeled by incubation with 4sU, followed by RNA extraction, TUC conversion treatment, and deep sequencing (Supplementary Fig. S1A). Data analysis using the GRAND-SLAM pipeline [44] revealed elevated T-to-C mutation frequency in all 4sU-labeled but not in unlabeled samples (Supplementary Fig. S1B), indicating successful 4sU incorporation and TUC conversion. In addition, principal component analysis confirmed that 4sU did not adversely affect gene expression, as evidenced by the close clustering of the unlabeled samples (DMSO) with the labeled samples at the corresponding time points of differentiation (Supplementary Fig. S1C).

**Figure 1.**
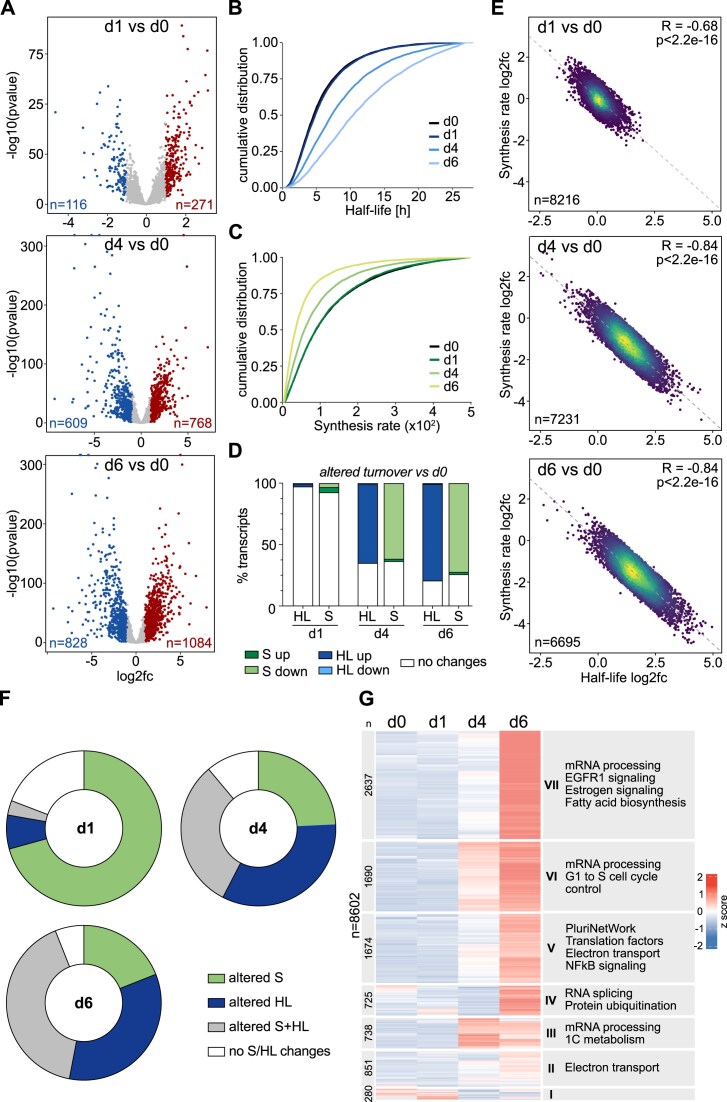
Gene expression changes during differentiation into NEC. (**A**) Volcano plots of differentially expressed genes (DEG) at d1, d4, and d6 of NEC differentiation versus mESC (d0). Colored dots indicate significantly regulated genes (|log_2_fc| > 1, FDR < 0.05). Cumulative distribution of ranked mRNA half-lives (**B**) and synthesis (**C**) in mESC (d0) and d1, d4, and d6 of NEC differentiation. (**D**) Proportion of transcripts exhibiting altered half-lives and/or synthesis at the indicated time points compared to mESCs (d0). HL/S values with absolute change |log_2_fc| > 1 and absolute region of practical equivalence |ROPE| > 0.45 were considered. HL, half-life; S, synthesis. (**E**) Correlation between S and HL of transcripts showing no significant changes (|log_2_fc| < 1, FDR > 0.05) of steady-state transcript levels at the indicated timepoints of differentiation compared to d0. Spearman correlation coefficients (R) and *P*-values are indicated. (**F**) Donut plots illustrating the proportion of DEGs exhibiting altered HL, S, or both at the indicated times of differentiation into NEC. (**G**) Heatmap of transcript HLs during NEC differentiation. Clusters were identified by k-means clustering. Significantly enriched GO term categories within the individual clusters are indicated.

We then examined the steady-state mRNA levels and found progressive changes over the course of differentiation (Fig. 1A and Supplementary Table S2). Key marker genes representing the neuroectoderm germ layer were induced, whereas crucial pluripotency genes or marker genes for endodermal and mesodermal germ layers were downregulated compared to mESCs confirming the efficiency of the differentiation protocol (Supplementary Fig. S2A).

Using the grandR software package [52], we estimated mRNA turnover parameters, i.e. synthesis (S) and HL. The data revealed high turnover rates for thousands of transcripts in mESCs reflected in short HL and high S rate (Fig. 1B and C; Supplementary Tables S3 and S4). The 10% most unstable transcripts in mESCs (*n* = 860) comprised many transcription factors and regulatory proteins (enriched GO terms: e.g. transcription coregulator activity, kinase regulator activity, chromatin DNA binding activity), while the most stable transcripts were enriched for GO categories translation regulator activity, unfolded protein binding, NAD binding, etc. (Supplementary Fig. S2B). These results are in good agreement with previous studies analyzing mESCs [60, 61]. When NEC differentiation was induced, the effect on RNA turnover was relatively modest at day 1 (d1). However, at day 4 (d4) and day 6 (d6), we observed widespread stabilization of transcripts and reduction of mRNA synthesis affecting thousands of transcripts (Fig. 1B and C; Supplementary Tables S3 and S4). Specifically, 64% and 78% of mRNAs had increased HL at d4 and d6, respectively, when compared to mESCs. 61% and 72% of transcripts showed reduced S (Fig. 1D). Transcript destabilization or increased synthesis, on the other hand, occurred very rarely at any differentiation time point (Fig. 1D). Furthermore, the data show that the proportion of transcripts exhibiting changes in one turnover parameter only (i.e. either synthesis or HL) was clearly larger during early differentiation, while at later time points, the majority of mRNAs showed simultaneous and opposing alterations of both parameters (Supplementary Fig. S2C). Notably, we observed a substantially greater number of transcripts displaying changes in turnover dynamics (HL and/or S) compared to those exhibiting changes in steady-state expression during differentiation. This was particularly pronounced for d4 and d6 of differentiation (Supplementary Fig. S2C), suggesting compensatory mechanisms balancing mRNA synthesis and stability. Indeed, analysis of all transcripts with unchanged steady-state levels but significantly altered HL and S relative to the ESC state, revealed a significant negative correlation between the two parameters (Spearman coefficient *R* = −0.63 at d1, *P*< .0001). This effect became stronger over time (*R* = −0.82 at d4, *P*< .0001; *R* = −0.82 at d6, *P*< .0001), indicating increasingly coordinated regulation of gene transcription and mRNA degradation during differentiation (Fig. 1E).

Focusing on transcript dynamics of genes with altered steady-state RNA levels upon differentiation induction compared to ESCs (DEGs), we found that 70% of DEGs exhibited altered S at d1 of differentiation, while only 7% showed altered HL. By contrast, at differentiation d4, most DEGs had altered HL, and at d6, the majority of DEGs showed simultaneous S and HL changes (Fig. 1F and Supplementary Fig. S2C). These observations suggest that the more immediate changes in steady-state transcript levels in response to signals triggering the exit from the stem cell state are governed by modulation of transcript synthesis rather than mRNA stability. In contrast, at later differentiation stages mechanisms regulating transcript stability become more important.

To determine whether there are differences in the timing of mRNA stabilization/destabilization among distinct groups of genes, cluster analysis based on HL values of individual transcripts was performed. Among the seven identified clusters, transcripts in clusters 3 and 6, which exhibited substantial stabilization as early as d4, were enriched in functional categories associated with mRNA processing, cell cycle control, pluripotency and differentiation, and one-carbon-metabolism (Fig. 1G). Transcripts in clusters 4, 5, and 7, which showed strong stabilization later in differentiation (d6), were enriched for functions related to RNA metabolism (processing, translation, splicing), as well as mRNAs involved in signaling, fatty acid biosynthesis, electron transport, and protein ubiquitination. An exception was the small cluster 1, which contained transcripts that became destabilized during differentiation. Although no significantly enriched functional categories were detected in this cluster, it contained transcripts encoding glutathione peroxidase (Gpx4). Notably, Gpx1, a close homolog of Gpx4, was previously reported to be required for stem cell self-renewal and to be quickly degraded at the onset of ESC differentiation [62].

Taken together, these results reveal that ESC differentiation affects the steady-state transcript levels of hundreds of genes by altering either mRNA synthesis or stability or both. Remarkably, a large fraction of the changes in mRNA turnover dynamics did not directly translate into altered transcript levels. This indicates a shift in gene regulatory mechanisms that precedes steady-state changes and/or mechanistically distinguishes different cell states by triggering transcript buffering, i.e. the compensatory adjustment of mRNA synthesis and decay rates. This phenomenon has been observed in various contexts, such as response to oxidative or DNA damaging stress or upon inactivation of key components of transcription or decay machineries [63–65]. Our data now provide a comprehensive view of the extent of transcript buffering that occurs during early mESC differentiation.

### Impact of Nsun2 on gene expression upon differentiation

Several reports have implicated mRNA modification by m^5^C in transcript stabilization [23–25]. Given the observed changes in mRNA stability during later stages of mESC differentiation into NEC, we investigated the role of Nsun2 as the major m^5^C mRNA methyltransferase in regulating mRNA turnover dynamics during differentiation. To this end, we inactivated the *Nsun2* gene in mESCs using CRISPR/Cas9. Targeting the catalytically important cysteine 321 (Cys321) resulted in short deletions in both alleles leading to premature stop codons and abolition of protein production (Supplementary Fig. S3A–C). Consequently, PCR-mediated bisulfite analysis of an established Nsun2 substrate tRNA (tRNA^Asp^) confirmed the loss of methylation at positions C47 and C48, whereas the methylation at position C38, which is targeted by the m^5^C methyltransferase Trdmt1, was preserved (Supplementary Fig. S3D). Knockout of Nsun2 did not affect mESC viability (Supplementary Fig. S3E), nor did it have an impact on cell cycle progression in mESCs or at d4 of NEC differentiation (Supplementary Fig. S3F and G).

**Figure 2.**
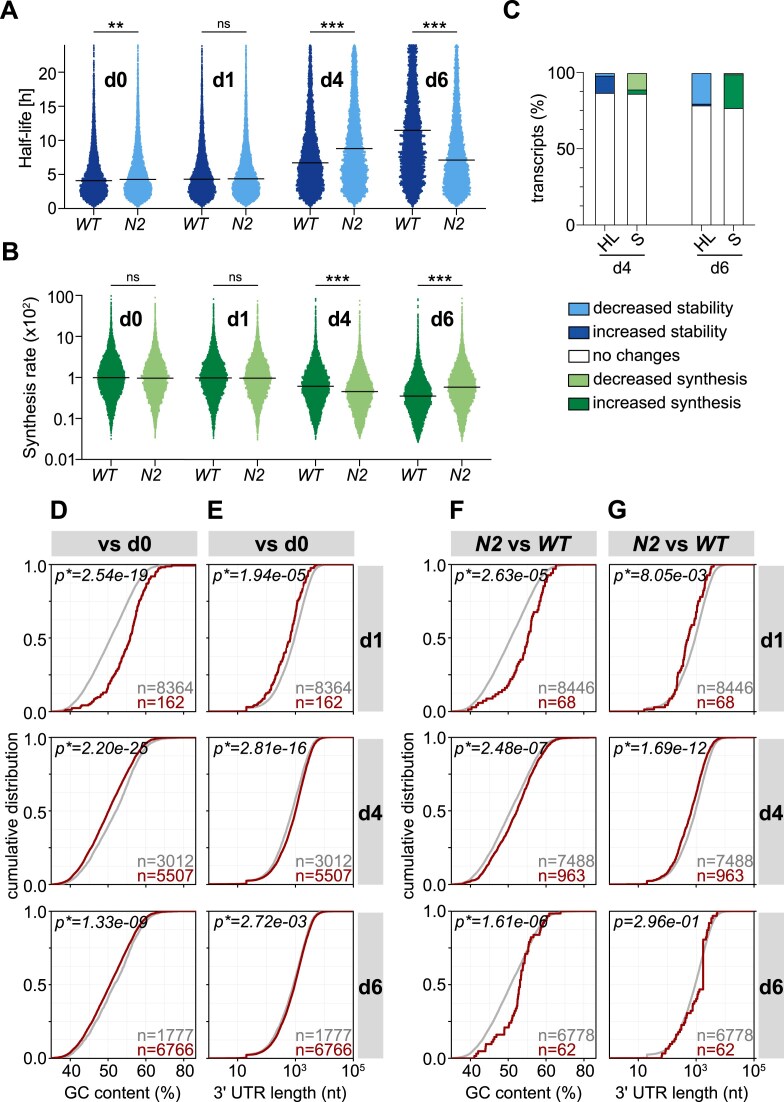
Loss of Nsun2 affects RNA turnover dynamics during mESC differentiation. (**A**, **B**) Beeswarm plots of mRNA half-lives (HL; A) or synthesis (S; B) in WT and *Nsun2^−/−^* (*N2*) mESCs (d0) and at d1, d4, and d6 of differentiation into NEC. Lines indicate median values. Statistical significance was determined by bootstrap analysis of median differences (ns, not significant—95% CI including zero, **99% CI not including zero, ***99.9% CI not including zero, 20 000 iterations). (**C**) Proportion of transcripts with increased/decreased HL in *Nsun2^−/−^* compared to WT cells at d4 and d6 of NEC differentiation. HL/S values with absolute change |log_2_fc| > 1 and absolute region of practical equivalence |ROPE| > 0.45 were considered. (**D**) GC content distribution of WT transcripts that show no changes in HL (gray) or increased HL (red) at the indicated time points of differentiation compared to d0. (**E**) Distribution of 3′UTR length of WT transcripts that show no changes in HL (gray) or increased HL (red) at the indicated time points of differentiation compared to d0. Statistical testing was done by Wilcoxon rank-sum test (*P< .05). Distribution of GC content (**F**) or 3′UTR length (**G**) in transcripts that show no changes in HL (gray) or increased HL (red) at the indicated time points in *Nsun2^−/−^*compared to WT cells.

TUC-seq analysis of RNA from *Nsun2^−/−^* cells revealed that in pluripotent mESCs, as well as at d1 of NEC differentiation, median mRNA synthesis rates and half-lives were very similar in WT and *Nsun2^−/−^* (Fig. 2A and B; Supplementary Tables S3 and S4). Upon further differentiation, however, we observed clear differences between the genotypes (Fig. 2A and B; Supplementary Fig. S4A and B). Specifically, at d4 and d6 of differentiation, 13% and 22% of all mRNA transcripts, respectively, exhibited differences in HL and/or S in *Nsun2^−/−^* compared to WT cells (Fig. 2C). Interestingly, we observed different patterns of dysregulation at early versus later differentiation time points. At d4 of differentiation, the majority of dysregulated mRNAs showed increased HL and reduced S in *Nsun2^−/−^* relative to WT cells. Conversely, at d6, median HL was lower and median S was higher in *Nsun2^−/−^* relative to WT cells (Fig. 2A and B; Supplementary Fig. S4A and B). In fact, in d6 cells, lack of Nsun2 interrupted the normally observed trend of mRNA stabilization/S reduction in the course of differentiation (Fig. 2A and B) and instead caused a shortening of median HL and increase in median S relative to the preceding time point (Fig. 2A and B). Given these considerable differences in RNA turnover dynamics between WT and *Nsun2^−/−^* cells, we expected to see concomitant changes in steady-state transcript levels. Surprisingly, however, only a few transcripts exhibited a more than two-fold increase in steady-state transcript levels at any given time point (Supplementary Fig. S4C).

To exclude the possibility that the differentiation protocol might have resulted in the observed differences of mRNA expression dynamics between *Nsun2^−/−^* and WT cells, we differentiated both cell lines into embryoid bodies (EBs) [66] and performed TUC-seq at 16 h (0.7 days) and 4 days after the induction of differentiation. Notably, the results showed time-dependent differences in HL and S between *Nsun2^−/−^* and WT cells that were consistent with those observed during NEC differentiation. Specifically, at d0.7, average HL was significantly longer and average S rate was significantly lower in *Nsun2^−/−^* cells compared to WT, while the opposite trend was observed for EBs at d4 of differentiation (Supplementary Fig. S4D and E; Supplementary Table S5).

In summary, these data demonstrate that although minimal changes in steady-state transcript levels were observed in *Nsun2^−/−^* ESCs and differentiating cells compared to WT cells within the time frame examined, the absence of Nsun2 strongly affected mRNA turnover dynamics in a differentiation-dependent manner. Similar to the compensatory changes of HL and S values observed during WT differentiation, the altered turnover dynamics caused by the loss of Nsun2 also appear to induce a buffering mechanism in order to maintain mRNA levels.

### Structural features associated with mRNA turnover dynamics

Previous studies have shown that certain structural features, such as transcript length or GC content, are correlated with mRNA stability [60, 67–69]. We investigated whether any of these features were associated with mRNA stability changes measured by TUC-seq during normal differentiation or with those changes occurring due to the loss of Nsun2 function. We first analyzed GC content and transcript length in all transcripts in WT cells that showed increased HL upon NEC differentiation relative to the ESC state. A comparison with transcripts that showed no changes in HL revealed that the relatively small subset of stabilized transcripts at d1 of differentiation had significantly higher GC content and shorter 3′UTR length than all other transcripts (Fig. 2D and E). In contrast, at d4 and d6 of differentiation, when the vast majority of transcripts had undergone stabilization, higher GC content and shorter 3′UTRs were detected in the remaining group of mRNAs with unaltered HL (Fig. 2D and E).

We next asked if such a correlation also existed for transcripts displaying HL changes caused by the loss of Nsun2 function. The results show that whenever transcripts were more stable in *Nsun2^−/−^* relative to WT cells, their GC content was significantly higher compared to the transcripts that were unaffected by loss of Nsun2 (Fig. 2F). Of note, we found that transcripts that exhibited decreased stability in *Nsun2^−/−^* compared to WT cells also displayed higher GC content relative to transcripts unaffected by *Nsun2* deletion (Supplementary Fig. S5A). Likewise, significantly shorter 3′UTR was associated with transcripts that were stabilized in *Nsun2^−/−^* compared to WT cells at d1 and d4 of differentiation, while such correlation was not observed for the small group of stabilized transcripts at d6 of differentiation (Fig. 2G). Transcripts that showed destabilization upon loss of Nsun2 at d1 and d6 of differentiation also exhibited significantly shorter 3′UTR length (Supplementary Fig. S5B).

Collectively, these findings indicate that there is a correlation between mRNA stability features, such as GC content and 3′UTR length, and the timing of HL regulation during normal ESC differentiation. This seems to be particularly true for the early response to differentiation signals. Interestingly, transcripts with elevated GC content and shorter 3′UTR also appear to be more susceptible to the loss of Nsun2 function because they tended to exhibit alterations in their turnover dynamics.

### Effect of mRNA methylation on RNA dynamics changes

Several studies suggested that m^5^C in mRNA contributes to transcript stabilization [23–25]. Therefore, a straightforward explanation for the relative reduction of global mRNA stability observed in *Nsun2^−/−^* cells, specifically at d6 of NEC differentiation, would be loss of m^5^C from the affected transcripts. However, it is more challenging to rationalize the observed stabilization of some mRNAs in *Nsun2^−/−^* cells relative to WT at d4 of differentiation. Since the current assumption is that m^5^C stabilizes transcripts, the mRNAs stabilized in *Nsun2^−/−^* cells should display relatively higher methylation levels than in WT cells. As Nsun2 function is absent in *Nsun2^−/−^* cells, this hypothesis can only be supported if other RNA methyltransferases would compensate for the loss of Nsun2 function.

**Figure 3.**
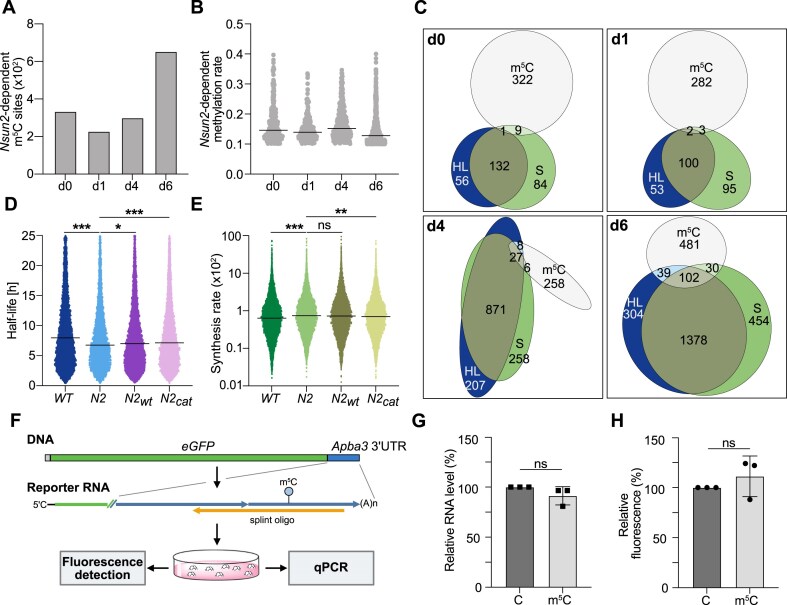
Absence of widespread cytosine methylation in transcripts with altered mRNA turnover dynamics in *Nsun2^−/−^* cells. (**A**) Nsun2-dependent methylation sites in ESC (d0) and at d1, d4, and d6 of NEC differentiation determined by BS-seq. (**B**) Methylation rates of Nsun2-dependent sites in undifferentiated (d0) and differentiated (d1, d4, d6) cells. Median values are indicated by a line. Wilcoxon test was performed to determine statistical differences (****P< .0001; ns, not signifiant). (**C**) Venn diagrams depicting the interrelationship between Nsun2-dependent methylated transcripts (m^5^C) and transcripts with altered HL and S values in *Nsun2^−/−^* compared to WT mESCs (d0) and during NEC differentiation (d1, d4, d6). HL/S values with absolute change |log_2_fc| > 1 and absolute region of practical equivalence |ROPE| > 0.45 were considered. Beeswarm plots of mRNA half-lives (**D**) or synthesis (**E**) in WT, *Nsun2^−/−^* (*N2*) cells, as well as in *Nsun2^−/−^* cells expressing either WT Nsun2 (N2_WT_) or catalytically inactive Nsun2 (N2_cat_). Cells were differentiated into the NEC lineage for 4 days. Note that the actual differentiation stage in this experiment appears to be later than d4 differentiation shown in Fig. 2 (see Supplementary Note S1 and Supplementary Fig. S7A and B for further details). Lines indicate median values. Statistical significance was determined by bootstrap analysis of median differences (ns, not significant—95% CI including zero, *95% CI not including zero, **99% CI not including zero, ***99.9% CI not including zero, 20 000 iterations). (**F**) Schematic depiction of a reporter RNA construct and experimental approach for analyzing the functional impact of m^5^C in the 3′UTR of the *Apba3* mRNA. (**G**) qPCR analysis of the relative transcript levels of *Cap-eGFP-Apba3-poly(A)* containing either a methylated or an unmethylated cytosine at position 1989 after transfection into HEK293T cells, relative to cotransfected *fLUC* mRNA and *GAPDH*. Mean ± SD of three independent experiments is shown. Unpaired *t*-test was used to determine statistical significance (ns, not significant). (**H**) Translation efficiency of the *Cap-eGFP-Apba3-poly(A)* reporter RNA was tested by measuring eGFP fluorescence in HEK293T cells. Mean ± SD of three independent experiments is shown. An unpaired *t*-test was used to determine statistical significance (ns, not significant).

To address these possibilities, we determined m^5^C in mRNA by bisulfite sequencing (BS-seq) using an improved protocol and analysis pipeline [41] that minimizes the calling of false positive methylation sites, which is a common caveat with BS-seq [8]. The results revealed 333 Nsun2-dependent sites in ESCs, 227 sites on d1, 299 sites on d4, and 652 Nsun2-dependent sites on d6 of NEC differentiation (Fig. 3A, Supplementary Table S6). The majority of sites were methylated at rates below 20% (Fig. 3B and Supplementary Table S6). We then examined the overlap between transcripts containing Nsun2-dependent m^5^C sites with those showing altered mRNA turnover dynamics (HL, S, or both) in *Nsun2^−/−^* compared to WT cells. Overlap was minimal, particularly in mESCs (Fig. 3C), and peaked at only ∼7.5% at d6 of differentiation (Supplementary Fig. S6A). Analysis of Nsun2-independent m^5^C sites showed that on d4 of differentiation, when RNA stability was increased in *Nsun2^−/−^* compared to WT cells, still only 6.9% of the affected transcripts carried m^5^C sites (Supplementary Fig. S6B and C). These results do not support the hypothesis that compensatory methylation by other methyltransferases was responsible for the observed stabilization of transcripts at d4 of differentiation in *Nsun2^−/−^* compared to WT cells. Instead, they suggest that the changes in RNA turnover dynamics observed in *Nsun2^−/−^* mutant cells were caused by loss of methylation-independent functions of the protein.

This conclusion was further substantiated by experiments involving the ectopic expression of an Nsun2 protein harboring a cysteine to glycine mutation at position 321 (C321G), which impairs catalytic activity (N2_cat_; Supplementary Fig. S7A) [12]. Expression of N2_cat_ in an *Nsun2^−/−^* background partially rescued differences in HL and S observed in *Nsun2^−/−^* compared to WT cells (Fig. 3D and E). Notably, in these experiments (Supplementary Fig. S7B and C), expression of WT Nsun2 (N2_wt_) in the *Nsun2^−/−^* background did not rescue the altered expression dynamics either (Fig. 3D and E), which was likely due to the very low levels of ectopically expressed N2_wt_ protein (Supplementary Fig. S7D and E; see Supplementary Note S1 for a detailed discussion).

Finally, we directly examined the potential impact of m^5^C on mRNA stability using an individual mRNA as an example. To this end, the *Apba3* mRNA was selected, which displayed increased HL in *Nsun2^−/−^* cells and carried an Nsun2-dependent m^5^C at position 1989 (3′UTR of transcript variant 1, sequence context: m^5^CAGGG, Supplementary Table S6). An RNA harboring the sequence encoding eGFP fused to the 3′UTR of *Apba3* containing either methylated or unmethylated C1989 was generated by splint ligation (Fig. 3F) and transfected into HEK293T cells, followed by mRNA stability and protein translation analysis. The results showed that neither mRNA stability nor eGFP protein production were significantly affected by the presence or absence of the m^5^C in the 3′UTR (Fig. 3G and H).

In summary, these results strongly suggest that direct methylation at cytosines in mRNA is unlikely to explain the observed changes in mRNA turnover dynamics in transcripts of *Nsun2*-mutant cells.

### Effects of Nsun2 loss on tRNA integrity in mESCs and differentiating cells

**Figure 4.**
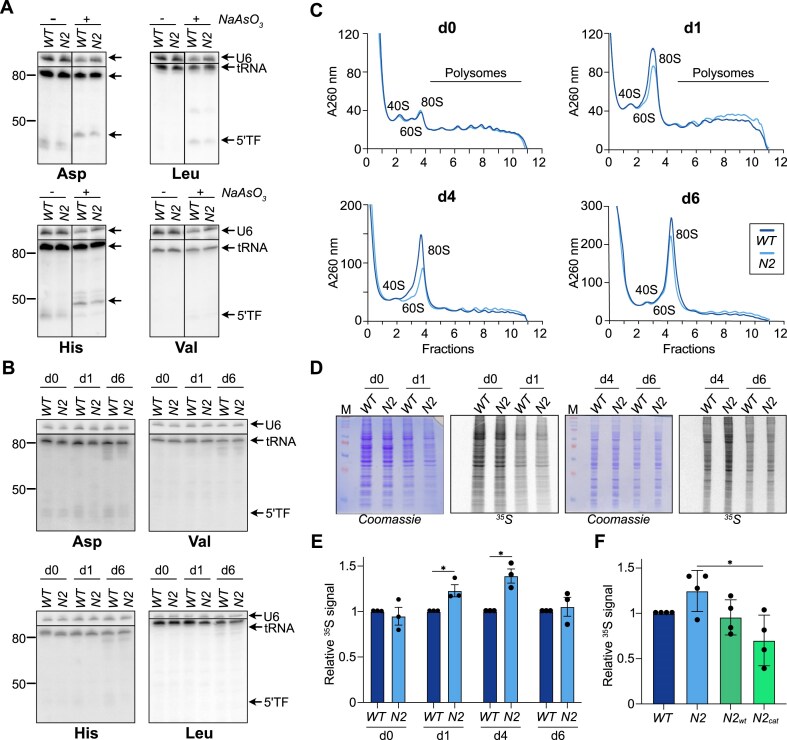
tRNA integrity and protein synthesis in differentiating *Nsun2^−/−^* cells. (**A**) Northern blot analysis to detect 5′tRFs (5′TF) of the indicated tRNAs under oxidative stress conditions. WT and *Nsun2^−/−^* ESCs (WT,*N2*) were exposed to 0.5 mM NaAsO_3_ for 90 min or left untreated. ^32^P-labeled probes complementary to the 5′ region of the indicated tRNAs were used for detection. tRNA_Asp_ and tRNA_Leu_ were detected on the same membrane one after the other. tRNA_His_ and tRNA_Val_ were detected on a different membrane (re-probed). U6 RNA was used as loading control. (**B**) Northern blots were performed at the indicated timepoints of differentiation with the probes described in panel (A), except that separate membranes were used for each tRNA. (**C**) Polysome profiles of WT and *Nsun2^−/−^* cells at the indicated time points of NEC differentiation. Areas corresponding to the polysome fractions, 40S, 60S, and 80S ribosomes are indicated. (**D**, **E**) Analysis of translation efficiency by metabolic ^35^S-methionine labeling. Labeling was performed for 30 min, followed by protein extraction, 10% SDS–PAGE, and radiography. (**D**) Representative images of Coomassie Brilliant Blue stained gels and corresponding radioactivity recordings are shown. (**E**) Signals were quantified by ImageJ and normalized against total protein (Coomassie staining). Mean ± SD of three independent experiments is shown. Statistical significance (*P< .0332) was tested by unpaired *t*-test. Only significant comparisons are shown. (**F**) Same as in panels (D) and (E) for WT, *Nsun2*^−/−^, and *Nsun2^−/−^* cells expressing WT (N2_wt_) or catalytically inactive Nsun2 (N2_cat_) that were subjected to NEC differentiation for 4 days. Mean ± SD of four experiments is shown. Statistical significance (**P< .008) was tested by one-way ANOVA. Only significant comparisons are indicated.

Nsun2 is best known for its role in the methylation of cytosines predominantly located in the variable loop of different tRNAs [8]. This modification has been shown to protect tRNAs from hydrolytic cleavage under certain conditions and in certain cell types [20, 21]. In order to investigate whether altered mRNA expression dynamics in *Nsun2^−/−^* cells might be indirectly linked to aberrant tRNA fragmentation, we examined the production of 5′ tRNA fragments (5′tRFs) in WT and *Nsun2^−/−^* mESCs and in differentiating cells. To serve as a positive control for 5′tRF production, mESCs were treated with sodium arsenite [70]. Using northern blot analysis of total RNA extracted from arsenite-treated and control mESCs, we found that although the production of 5′tRFs from various known Nsun2 target tRNAs (tRNA^Asp^, tRNA^Val^, tRNA^His^, tRNA^Leu^) increased in response to arsenite-induced stress, there was no difference between WT and *Nsun2^−/−^* cells (Fig. 4A). We then subjected both cell lines to NEC differentiation and measured tRNA and 5′tRF levels at d1 and d6 of differentiation. The data showed that, with the exception of fragments derived from tRNA^Asp^, 5′tRFs were barely detectable. Again, we did not observe appreciable differences between WT and *Nsun2^−/−^* cells (Fig. 4B). These findings indicated that the loss of Nsun2 does not alter 5′tRF production or change the levels of several Nsun2-target tRNAs in mESCs and during NEC differentiation, at least within the detection limits of northern blot analysis. Therefore, changes at the tRNA level were unlikely to account for altered mRNA turnover dynamics in *Nsun2^−/−^* cells.

### Effect of Nsun2 loss on global translation in differentiating stem cells

Given the unlikelihood that cytosine-5 methylation of mRNA caused the observed differences in RNA turnover dynamics between WT and *Nsun2^−/−^* cells combined with the finding that tRNA fragmentation was not significantly affected by loss of Nsun2, we conducted a more detailed analysis of those mRNAs that exhibited no change in HL between the ESC state and d4 of differentiation in WT but displayed increased stability in *Nsun2^−/−^* cells. Overrepresentation analysis of WikiPathways revealed significant enrichment of functional categories, including translation factors, mRNA processing, and amino acid metabolism, suggesting that translation might be affected by the loss of Nsun2 (Supplementary Fig. S8). Therefore, we investigated translation efficiencies in WT and *Nsun2^−/−^* mESCs and at d1, d4, and d6 of NEC differentiation using polysome profiling. The results revealed no differences in translation efficiencies in ESCs from both genotypes. In contrast, upon differentiation toward the neuroectodermal lineage, *Nsun2^−/−^* cells showed a decrease in the monosome fraction and a slight increase in the polysome fraction compared to WT cells suggesting that the absence of Nsun2 in differentiating cells promoted translation (Fig. 4C).

Consistent with this observation, metabolic labeling experiments using ^35^S-methionine revealed a moderate (∼25%) yet significant increase of newly synthesized proteins at d1 and d4 of NEC differentiation in *Nsun2^−/−^* cells, while no significant differences were detected in ESCs and at d6 of differentiation (Fig. 4D and E). Interestingly, ectopic expression of both N2_wt_ and N2_cat_ was sufficient to revert translation to WT levels in cells at d4 of NEC differentiation (Fig. 4F) even though N2_wt_ was expressed at low levels. These observations further suggest a methylation-independent function of Nsun2 in the regulation of translation.

In conclusion, the absence of Nsun2 during mESC differentiation resulted in changes of mRNA turnover dynamics and in the stimulation of translation at early differentiation time points. The molecular mechanisms driving these effects do not seem to involve direct cytosine methylation of mRNAs or methylation-dependent alterations in tRNA levels and integrity, pointing to a previously unrecognized, methylation-independent function of Nsun2.

## Discussion

Pluripotency and differentiation of ESCs are governed by a complex network of molecules regulating highly coordinated gene expression programs at multiple levels including chromatin, transcription, and posttranscriptional processes [71–74]. In this work, we studied RNA turnover dynamics, i.e. mRNA half-lives and synthesis rates, in pluripotent and differentiating mESCs and addressed the potential role of the conserved RNA cytosine methyltransferase Nsun2 in these processes.

### Differentiation-dependent alterations in mRNA turnover dynamics

Our data demonstrated high turnover of mRNA in pluripotent ESCs. This result correlates well (Spearman coefficient *r* = 0.76, *P*< .0001) with a previous study in ESCs [61], which used a different RNA labeling strategy (long pulse-chase versus short pulse in our study) and employed SLAM-seq as an alternative 4sU-to-C conversion method. The similarity of the primary data indicates that TUC-seq is well suited to examine RNA turnover dynamics. Furthermore, we show that differentiation is accompanied by substantial global stabilization of mRNAs and a concurrent decrease in mRNA synthesis. Notably, very few studies have examined mRNA turnover dynamics in the course of ESC differentiation. One previous study reported on a similar trend toward mRNA stabilization when ESCs were differentiated using retinoic acid. Interestingly, this trend was not observed when differentiation was induced by LIF removal [60]. However, it is important to note that the mRNA HL measurements in this study were conducted using transcriptional shutoff (actinomycin D treatment), a method known to induce a cellular stress response that can affect mRNA stability [67, 75].

What might be the cause of enhanced mRNA stability during differentiation? It has been reported that mRNA degradation rates correlate with cell cycle length [76, 77]. Therefore, as the duration of the cell cycle is shorter in pluripotent ESCs than in differentiating cells [78, 79], at least a proportion of the observed mRNA stabilization may be linked to increased cell cycle length. Alternatively, or in addition, specific structural characteristics of transcripts, such as GC content or 3′UTR length, may render certain mRNAs particularly susceptible to rapid response to differentiation signals by HL/S changes [60, 67–69]. Indeed, we found a significant enrichment of transcripts with comparatively higher GC content and shorter 3′UTR length among the stabilized transcripts at the initial time points of NEC differentiation, especially when the number of affected mRNAs was still low. By contrast, correlations were moderate or even inverse at later time points of differentiation when thousands of transcripts displayed altered turnover dynamics. Thus, certain structural features appear to render transcripts especially sensitive toward stability changes at the onset of differentiation.

Differentiation, as widely recognized, caused significant alterations in steady-state levels of many mRNAs. Our transcript turnover analysis now shows that the mechanisms underlying these changes cause distinct alterations in S and/or HL depending on the timepoint during differentiation. During the initial phase (d1), steady-state changes were accompanied primarily by the alteration of synthesis rate rather than HL. These results confirm the findings by Freimer *et al.*, who showed that nascent transcription is the predominant driver of mRNA changes during the differentiation of naïve ESCs to formative epiblast-like cells [80]. Furthermore, mRNA synthesis rather than stability was found to constitute the primary response to external cues in other biological systems. This includes the response to hypoxia or lipopolysaccharide stimulation [81–83]. However, our data also show that at later differentiation stages, HL rather than S changes affected a larger proportion of DEGs, and eventually, most DEGs showed changes in both S and HL.

**Figure 5.**
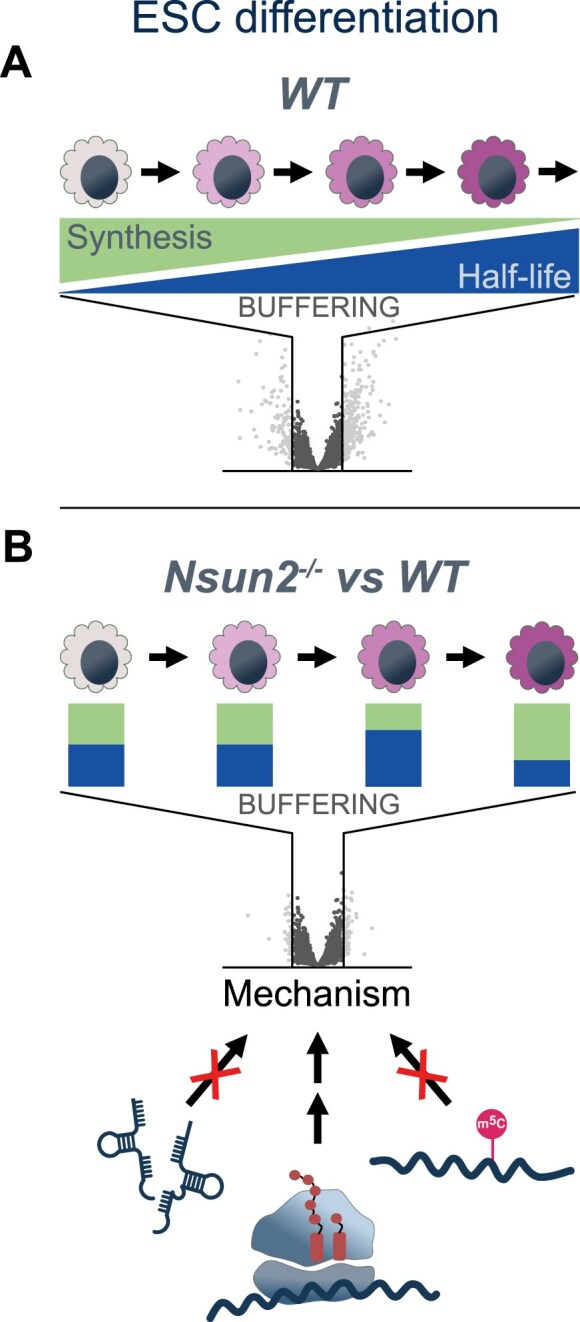
Model of mRNA turnover dynamics during ESC differentiation. (**A**) During differentiation, the steady-state levels of many transcripts change, yet the majority of mRNAs remain constant. However, the underlying turnover dynamics of these unchanged transcripts are altered as differentiation progresses. A buffering mechanism preserves transcript levels by increasing mRNA stability while reducing synthesis rates. (**B**) Knockout of *Nsun2* elicits changes in mRNA half-life (blue) and synthesis (green) compared to WT cells, resulting in higher mRNA stability in early differentiating cells and lower stability at later time points. Similar to the process observed during differentiation, these changes are buffered by compensatory adjustments in turnover parameters, thereby maintaining steady-state mRNA levels. Notably, the mechanism underlying these changes does not involve cytosine methylation in mRNA or alterations in tRNA abundance or integrity. Instead, Nsun2 may indirectly influence mRNA stability through a catalysis-independent effect on translation.

Interestingly, we found that during ESC differentiation into NEC, alterations in turnover dynamics were largely uncoupled from changes in steady-state RNA levels affecting considerably more transcripts. Moreover, HL and S changed in opposite directions on the same transcript providing evidence for a buffering mechanism during ESC differentiation (Fig. 5A). Transcript buffering, i.e. the compensatory adjustment of RNA synthesis and decay rates to maintain mRNA levels, has been described by several studies as a consequence of interfering with proteins involved in either transcription or mRNA decay [64, 65, 84]. One of the earliest examples of this phenomenon in mammalian cells was reported in a study of the general transcription factor TFIIH. Specifically, upon deletion of a regulatory component of TFIIH, the steady-state levels of most mRNAs remained largely unaffected, despite a severe reduction in nascent RNA production. This unexpected finding led the authors to propose that the negative impact on transcription must be counterbalanced by the cell through changes in mRNA stability [85]. Coordinated regulation of S and HL is thought to involve the crosstalk between transcription in the nucleus and mRNA degradation mostly occurring in the cytoplasm. Although the molecular mechanisms are not yet fully understood, studies in *Saccharomyces cerevisiae* have identified several RNA-binding proteins with dual roles in regulating both transcription and mRNA degradation, thereby coordinating these essential processes of gene expression [64, 65, 84, 86–90]. An interesting focus of future studies might be to determine which factors contribute to differentiation-associated RNA buffering and how these processes are regulated as differentiation progresses.

### Role of Nsun2 in the regulation of mRNA turnover dynamics

An important finding of our study is that the loss of Nsun2 also triggered a transcript buffering mechanism in cells differentiating into NEC (Fig. 5B). We found that a considerable number of transcripts exhibited altered stability and synthesis in the absence of Nsun2, while steady-state mRNA levels were barely affected. This effect was particularly evident at d4 and d6 of differentiation, whereas only few genes showed changes in mESCs and at d1 of differentiation.

As Nsun2 is a highly conserved protein involved in RNA modification and RNA metabolism, an involvement in transcript buffering mechanisms is perhaps not very surprising, given that this phenomenon has been observed following disruption of a variety of factors involved in the same process [64, 65, 84, 86–90]. There are several potential mechanisms through which Nsun2 could influence RNA turnover dynamics (Fig. 5B).

- Nsun2 could stabilize mRNA by direct methylation, as previously proposed, resulting in the recruitment of m^5^C reader proteins [23, 24]. However, our findings indicate that only a small fraction of transcripts with altered dynamics carries Nsun2-dependent or -independent methylation sites. In contrast, the majority of m^5^C-bearing transcripts showed no changes in stability and/or synthesis. Furthermore, the stoichiometry of m^5^C at specific positions in any given mRNA was rather low (<20%). Therefore, it is difficult to envisage that the loss of m^5^C methylation from mRNA would directly cause the observed phenotypes. Supporting this conclusion, our experiments showed that a reporter RNA containing either a fully methylated or unmethylated cytosine at a specific position did not exhibit altered stability in a cell culture model. Moreover, expressing a catalytically inactive N2_cat_ version partially rescued the mRNA stability differences observed in *Nsun2^−/−^* cells. Taken together, these results therefore strongly argue against the notion that direct methylation of mRNA by Nsun2 controls mRNA turnover dynamics in the context of mESC proliferation and differentiation into NEC.
- Nsun2 could affect mRNA dynamics indirectly through its established role as a tRNA modification enzyme. Nsun2-mediated methylation is thought to reduce stress-induced cleavage of tRNAs by angiogenin, thereby limiting the potential negative effects of the tRNA fragments on translation [20, 21]. However, in addition to the observation of increased rather than decreased translation in the absence of Nsun2 in differentiating cells, we found no evidence for altered tRNA fragmentation or tRNA levels, either in *Nsun2^−/−^* ESCs in response to sodium arsenite stress or during differentiation into NEC. Therefore, a tRNA methylation-related mechanism is also unlikely to explain the observed effects on mRNA turnover dynamics and translation.
- mRNA stability might be affected by differentiation defects caused by Nsun2. The finding that transcript stability increased steadily over time during NEC differentiation in WT cells could lend support to the hypothesis that transcript stabilization may be considered a marker of differentiation. To illustrate this notion, all but one of the transcripts that were stabilized from d1 to d4 of differentiation displayed further stabilization beyond d4 in WT cells. In stark contrast, *Nsun2^−/−^* cells failed to further stabilize the vast majority (91%) of mRNAs from d4 to d6 of differentiation. Instead, many transcripts exhibited again shorter HL at differentiation d6 compared to d4. Thus, the absence of Nsun2 might indeed induce a delay in or the stalling of differentiation at later stages of NEC differentiation. As a matter of fact, several reports showed that Nsun2 deletion inhibited lineage commitment or differentiation in various contexts [19, 20, 91, 92]. However, as most of these studies linked the differentiation defects to the accumulation of 5′tRNA fragments and thereby to reduced translation, the increase in translation in differentiating *Nsun2^−/−^* cells observed in our study argues against a similar mechanism in early ESC differentiation. Furthermore, the lack of significant differential gene expression, as demonstrated by the volcano plots in Supplementary Fig. S4C, along with the differentiation-dependent but genotype-independent clustering of RNA-seq samples after principal component and hierarchical clustering analyses (Supplementary Figs S1C and S7B), support the idea that WT and *Nsun2^−/−^* cells are not sufficiently different in their differentiation status to account for the observed changes in mRNA turnover dynamics.
- Nsun2 might affect transcript stability in a catalysis-independent fashion, for instance, through interactions with certain RNA-binding proteins that regulate mRNA turnover dynamics. A recent report found Nsun2 as a potential interaction partner of Caprin1 in ESCs. Although the functional link between the two proteins was not addressed, Viegas *et al.* showed that Caprin1 is required for mRNA degradation in early differentiation and that its knockdown resulted in widespread transcript stabilization [93], which is similar to our findings upon knockout of Nsun2. Further catalysis-independent functions of Nsun2 have been previously reported, such as restoring mitotic spindle integrity in a breast cancer cell line [12] or rescuing translation defects in *NSUN2*-defective human dermal fibroblasts [92].
- Finally, Nsun2 might directly affect translation, which in turn might impact on mRNA stability. Translation is known to influence transcript stability, even though the exact molecular mechanisms remain uncertain. The general view implies a positive correlation between mRNA stability and translation in different organisms and cell types including ESCs [80, 94, 95]. Accordingly, increased translation in the absence of Nsun2 could be responsible for the observed stabilization of many transcripts during early differentiation. It is worth noting, however, that the effects of Nsun2 depletion have been reported to differ across studies, resulting in varying outcomes with regard to translation. Some studies showed that translation is inhibited in the absence of Nsun2 in dermal cell types [21, 92], while reports from other systems concluded the opposite. For instance, embryonic fibroblasts from *Nsun2* knockout mice exhibited increased translation [96], and knockdown of *NSUN2* in HEK293T cells also resulted in upregulation of translation [33], suggesting that there might be tissue- and cell-type specific differences. In any case, the upregulation of translation during ESC differentiation could potentially explain the mRNA stability increase observed in *Nsun2* KO cells. However, the exact molecular nature of the potential inhibitory effect of Nsun2 on translation remains unclear at this stage, although there is accumulating evidence for a catalysis-independent function of Nsun2 in translation (this study, [92]).

In summary, by employing the minimally invasive and robust TUC-seq method, a comprehensive understanding of the mRNA turnover landscape in self-renewing mESCs and during early differentiation was obtained. Our findings provide strong evidence for the existence of a robust transcript buffering mechanism throughout differentiation that coordinates RNA synthesis and decay rates to maintain the abundance of hundreds of mRNAs. Additionally, we show that the loss of Nsun2 impacts the stability and synthesis rates of numerous mRNAs while upregulating translation during early differentiation. However, contrary to previous reports, our data do not support a significant role for m^5^C in mRNA in this process.

## Supplementary Material

gkaf995_Supplemental_Files

## Data Availability

The data underlying this article are available in the article and in its online supplementary material. TUC-seq and bisulfite sequencing data have been deposited at SRA (Sequence Read Archive) under BioProject PRJNA1185426.
