# Correction to ‘mRNA turnover dynamics are affected by cell differentiation and loss of the cytosine methyltransferase Nsun2’

**DOI:** 10.1093/nar/gkaf1375

**Published:** 2025-11-28

This is a correction to: Isabel Delazer, Ingo Bauer, Teresa Rummel, Kamila Nykiel, Dietmar Rieder, Magdalena Fickl, Valentin Tumler, Anna Razkova, Matthias R Schaefer, Thalissa Scheed, Matthias D Erlacher, Florian Erhard, Ronald Micura, Alexandra Lusser, mRNA turnover dynamics are affected by cell differentiation and loss of the cytosine methyltransferase Nsun2, Nucleic Acids Research, Volume 53, Issue 19, 28 October 2025, gkaf995, https://doi.org/10.1093/nar/gkaf995

This article was incorrectly categorized as a Standard research article. This was an editorial oversight.

The article should have been designated as a Breakthrough article, reflecting its significant contribution to the field.

This correction does not affect the content of the article. The online version has been updated to reflect the correct article category.

We apologize to the authors and readers for this error.

