# Supplementary material for: mRNA turnover dynamics are affected by cell differentiation and loss of the cytosine methyltransferase Nsun2
Source: Nucleic Acids Res. 2025 Oct 16;53(19):gkaf995. doi: 10.1093/nar/gkaf995 (PMC12529931; doi:10.1093/nar/gkaf995)
Supplement: gkaf995_Supplemental_Files [file gkaf995_supplemental_files.zip › Supplementary Material_26092025.pdf]

Supplementary Material

Supplementary Note S1……………………………………………………………………………………2

Supplementary Figures

Figure S1……………………………………………………………………………………………………3

Figure S2……………………………………………………………………………………………………4

Figure S3……………………………………………………………………………………………………5

Figure S4……………………………………………………………………………………………………6

Figure S5……………………………………………………………………………………………………7

Figure S6……………………………………………………………………………………………………8

Figure S7……………………………………………………………………………………………………9

Figure S8……………………………………………………………………………………………………10

Supplementary Note S1

Generation and characterization of ESCs expressing wildtype or catalytically inactive Nsun2 in an *Nsun2*$^{-/-}$ background

To characterize expression of the *Nsun2* transgenes in *N2*$_{WT}$ and *N2*$_{cat}$ ESCs, qPCR and western blot analyses were performed. To confirm functionality of the ectopically expressed proteins, PCR-mediated BS-seq of the Nsun2-target tRNA^{Asp} was conducted. Supplementary Figure S7D and E show that *N2*$_{cat}$ is expressed at a level that is comparable to endogenous *Nsun2* in ESCs, while *N2*$_{WT}$ exhibits substantially lower expression. Consistent with these observations, BS analysis revealed only partial rescue of m^{5}C at positions 47 and 48 of tRNA^{Asp} in *N2*$_{wt}$ cells, while no m^{5}C was detected for the cell line expressing catalytically inactive N2$_{cat}$ (Supplementary Figure S7A).
WT, *Nsun2*$^{-/-}$, *N2*$_{WT}$ and *N2*$_{cat}$ cell lines were subjected to NEC differentiation followed by metabolic labeling with 4sU and TUC-seq. To compare the actual differentiation stage of the cells (hereafter termed experiment 2) with that of cells of the earlier differentiation experiment (hereafter termed experiment 1), whose results are shown in most figures of the manuscript, cluster analysis of sequencing data from all samples was performed. To this end, all transcripts exhibiting significant regulation ($|\log2fc| > 1$, $p < 0.05$); DESeq2 likelihood ratio test) throughout differentiation were subjected to k-means clustering after z-score transformation. The heatmap in Supplementary Figure S7B reveals that all samples from experiment 2 (grey box in Supplementary Figure S7B) cluster in between d4 and d6 of experiment 1. Similarly, analysis of the cumulative distribution of half-lives in WT samples from the two experiments places the HL data from experiment 2 in between d4 and d6 of experiment 1 (Supplementary Figure S7C). Based on these findings, we conclude that the differentiation stage of the cells in experiment 2 corresponds to approximately day 5 (d5) of NEC differentiation in experiment 1.

The discrepancy between the differentiation stages in experiments 1 and 2 may be attributed to several factors, including the time gap between the experiments (approximately two years), differences in the individuals conducting the experiments, or a slightly higher passage number of the ESCs used in experiment 2.

Importantly, the relative comparisons between the samples within experiment 2 remain unaffected by these differences, as evidenced by the close clustering of the distinct genotypes.

Supplementary Figures

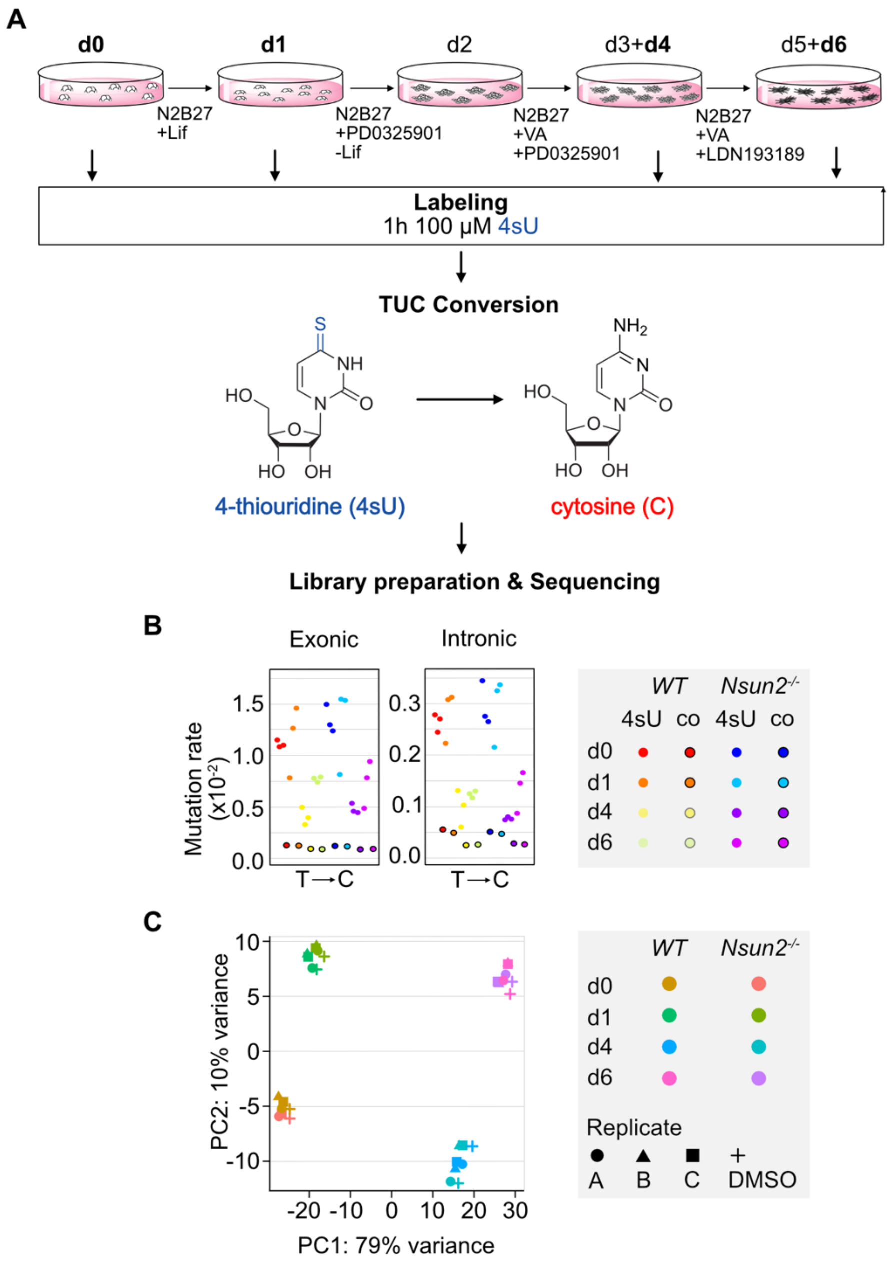

Supplementary Figure S1 (**A**) Schematic overview of the experimental approach for NEC differentiation, 4sU labeling and TUC-sequencing. (**B**) T-to-C mutation rates detected by TUC-seq in *WT* and *Nsun2*$^{-/-}$ cells at different time points of differentiation into NEC as indicated in the legend. 4sU labeled (4sU) and unlabeled samples (co) show clear differences with regard to T-to-C mutation rates in exonic and intronic reads. (**C**) Principal component analysis (PCA) of all TUC-seq samples reveals distinct clustering based on differentiation time point, while showing no clear separation according to genotype or labeling condition.

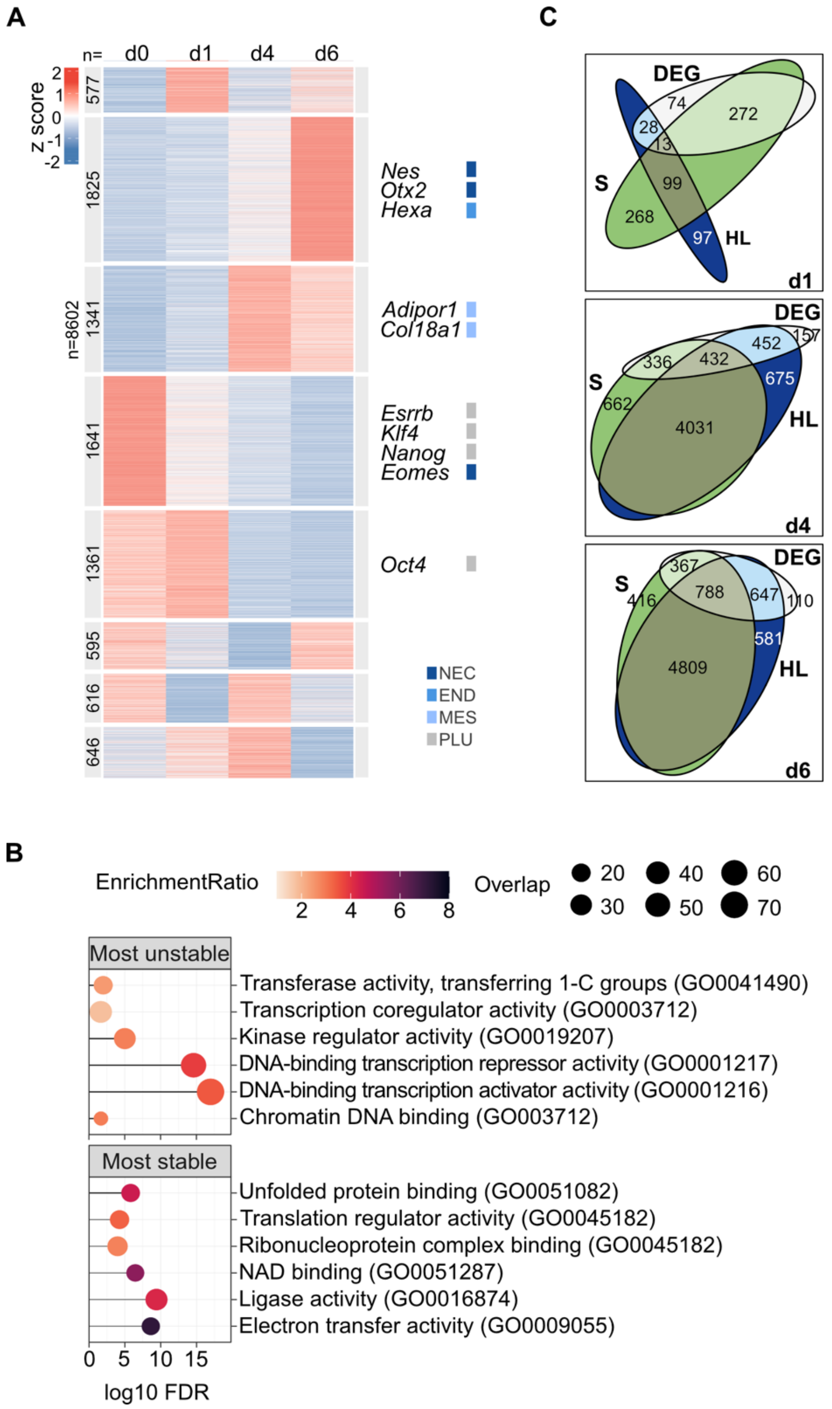

Supplementary Figure S2. Transcript synthesis and half-lives dynamically change upon induction of differentiation. (**A**) Heatmap of expression profiles during NEC differentiation. Example marker genes of pluripotency (PLU), meso- (MES), endo- (END) and neuroectodermal (NEC) germ layers are listed. (**B**) GO term over-representation analysis of the 10% most unstable (860) and 10% most stable (860) transcripts in *WT* ESCs. The six most significantly enriched categories are shown. Colors denote enrichment ratio, and circle size corresponds to the number of overlapping genes. GO term identifiers are shown in parentheses. (**C**) VENN diagram depicting the relationship between differentially expressed genes (DEGs) and transcripts with significantly altered HL and/or S at different timepoints of NEC differentiation (d0, d1, d4, d6). DEGs with |log2fc| > 1, FDR < 0.05, and HL/S values with absolute change |log2fc| > 1 and absolute region of practical equivalence |ROPE| > 0.45 were considered.

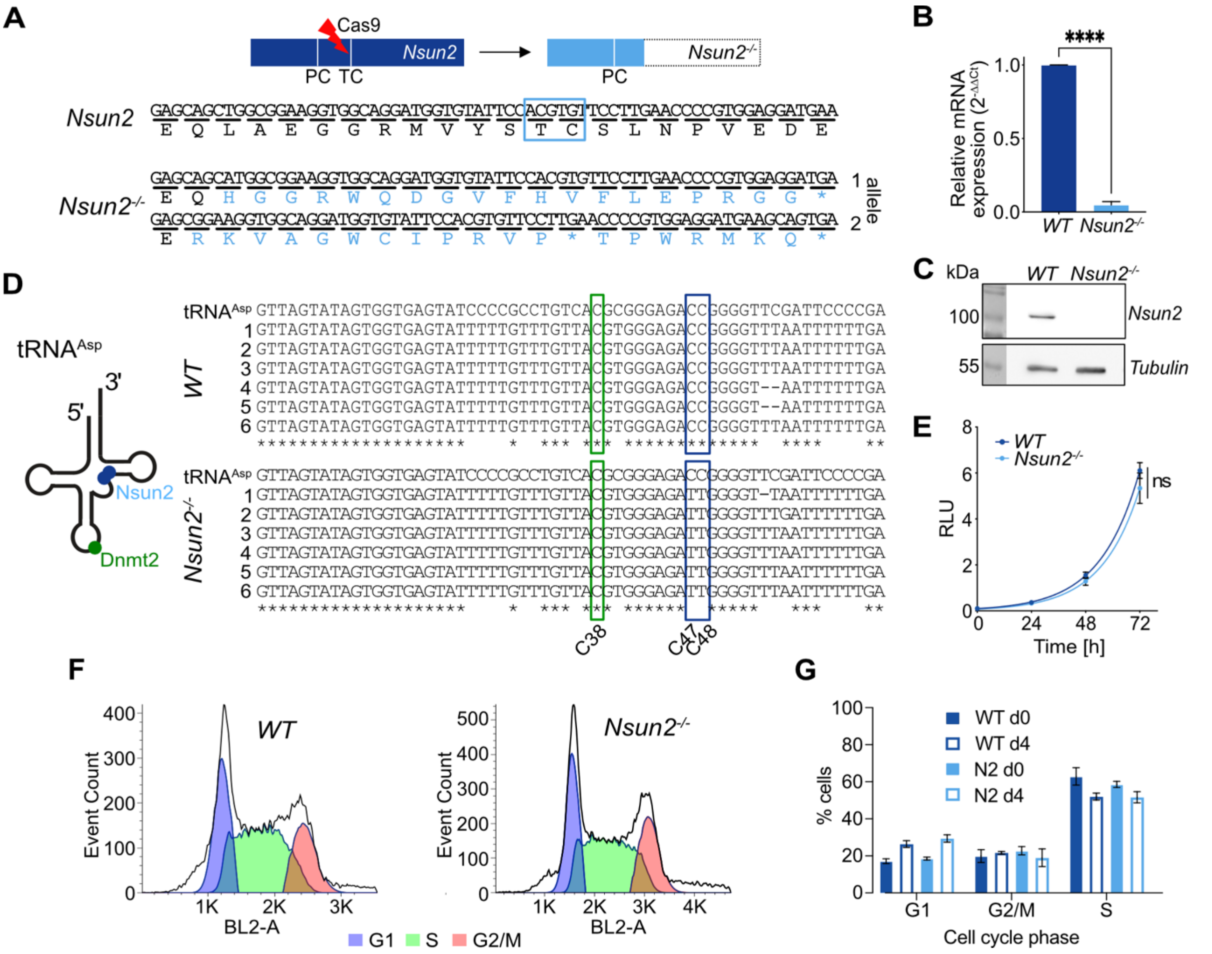

Supplementary Figure S3. Generation and characterization of an *Nsun2*$^{-/-}$ ESC line. (**A**) CRISPR/Cas9-mediated targeting of the catalytically important $T_{320}C_{321}$ motif (blue box) resulted in frameshift mutations on both alleles causing premature stop codons. Altered peptide sequence is indicated in blue. (**B**) Relative *Nsun2* expression in *WT* and *Nsun2*$^{-/-}$ cells was determined by RT-qPCR. $2^{-\Delta\Delta Ct}$ values were calculated with *TATA binding protein* (*TBP*) as the reference gene. Mean±SD is shown (unpaired t-test ****p<0.0001; n= 3). (**C**) Western blot of whole cell extracts from *WT* and *Nsun2*$^{-/-}$ ESCs were probed with α-Nsun2 antibodies. Tubulin was detected to control for equal loading. (**D**) PCR-mediated BS-seq of tRNA^{Asp} revealed absence of Nsun2-mediated methylation in *Nsun2*$^{-/-}$ cells, while Dnmt2-mediated methylation was preserved. Six sequences from individual tRNA clones from *WT* and *Nsun2*$^{-/-}$ cells are shown. Blue box, Nsun2-target sites C47, C48; green box, Dnmt2 target site C38. (**E**) Cell viability of *WT* and *Nsun2*$^{-/-}$ cells was determined every 24 h over a total period of 72 h using the CellTiterGlow 2.0 assay. Mean±SD of two experiments with three technical replicates each is shown. Statistical testing was done by unpaired t-test for the last timepoint (p<0.05). Ns, not significant. (**F**) Example FACS profiles of *WT* and *Nsun2*$^{-/-}$ cells at d4 of NEC differentiation. (**G**) Quantification of distribution of cell cycle phases in ESCs and d4 differentiated cells. Mean ±SD of three independent FACS experiments are shown. Statistical analysis was performed by unpaired t-test. No significant differences were detected.

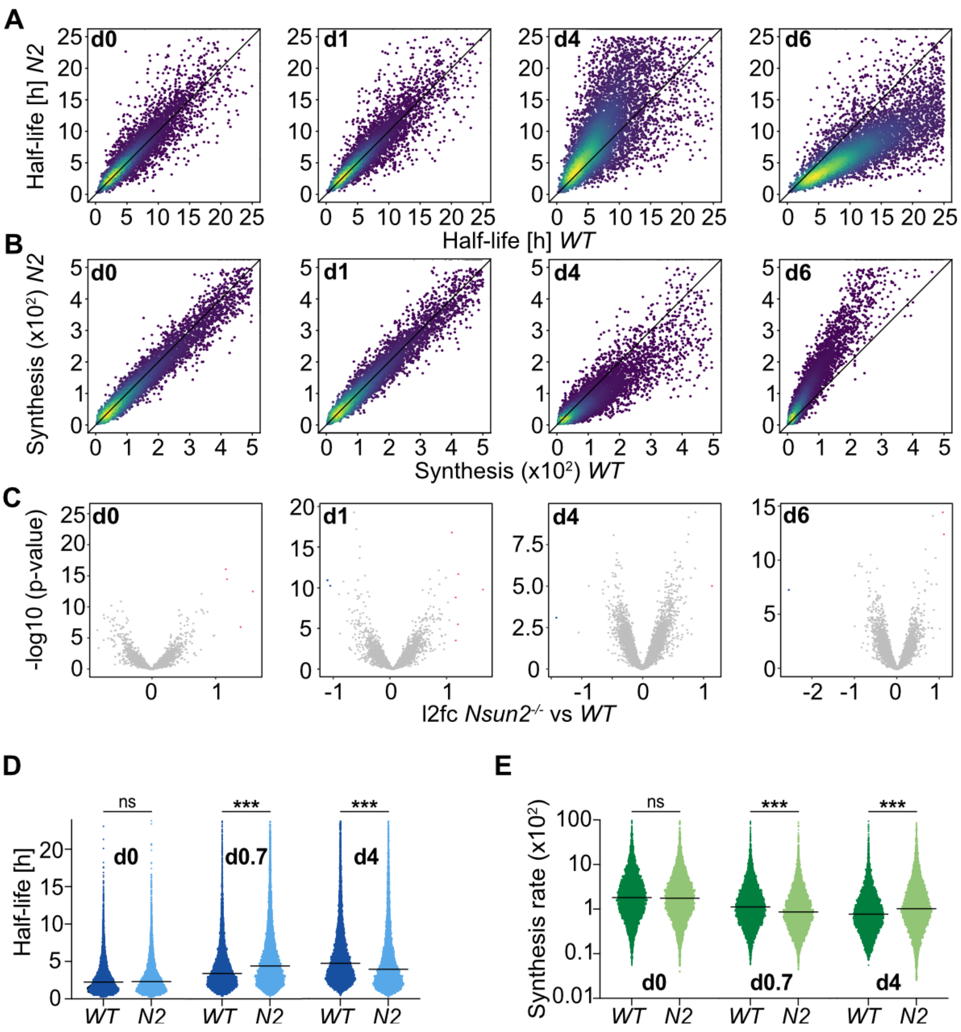

Supplementary Figure S4. Loss of Nsun2 affects mRNA turnover dynamics. (**A, B**) Half-lives (**A**) or synthesis rates (**B**) of all detected transcripts in *WT* vs *Nsun2*$^{-/-}$ (N2) cells at different time points of differentiation into NEC. (**C**) Volcano plots of mRNA steady-state levels in *WT* vs *Nsun2*$^{-/-}$ at different time points of NEC differentiation. Significance threshold was set to |log2fc| > 1 and FDR < 0.05. (**D, E**) Beeswarm plots of mRNA half-lives (**D**) and synthesis (**E**) in *WT* and *Nsun2*$^{-/-}$ ESCs (d0) and at d0.7, and d4 of differentiation into embryoid bodies. Lines indicate median values. Statistical significance was determined by bootstrap analysis of median differences (ns, not significant - 95% CI including zero, ***99.9% CI not including zero, 20,000 iterations).

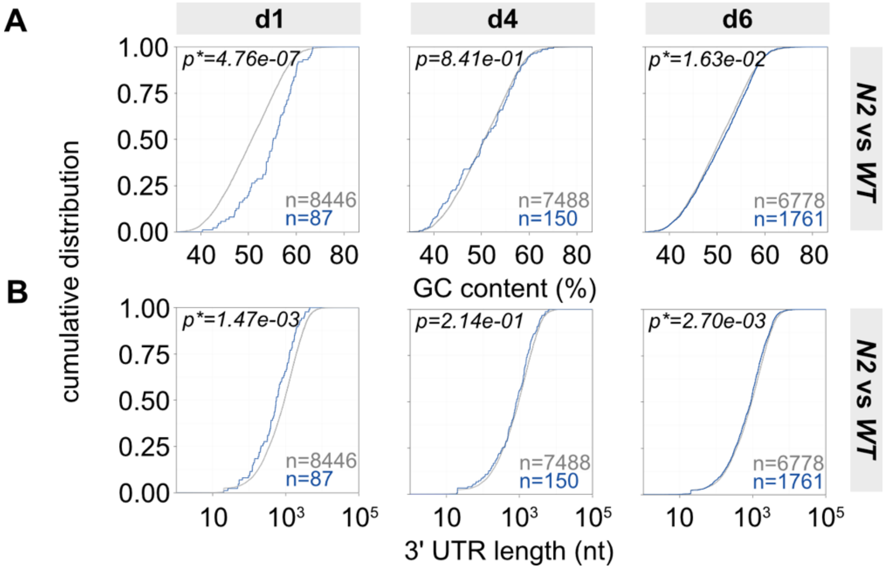

Supplementary Figure S5. Structural features associated with mRNA turnover dynamics. (**A, B**) GC content distribution (**A**) and 3'UTR length (**B**) of transcripts showing no changes in half-life (no; grey) or decreased half-life (down; blue) at the indicated time points of NEC differentiation in *Nsun2*$^{-/-}$ compared to *WT* cells. Statistical testing was done by unpaired t-test (*p<0.05).

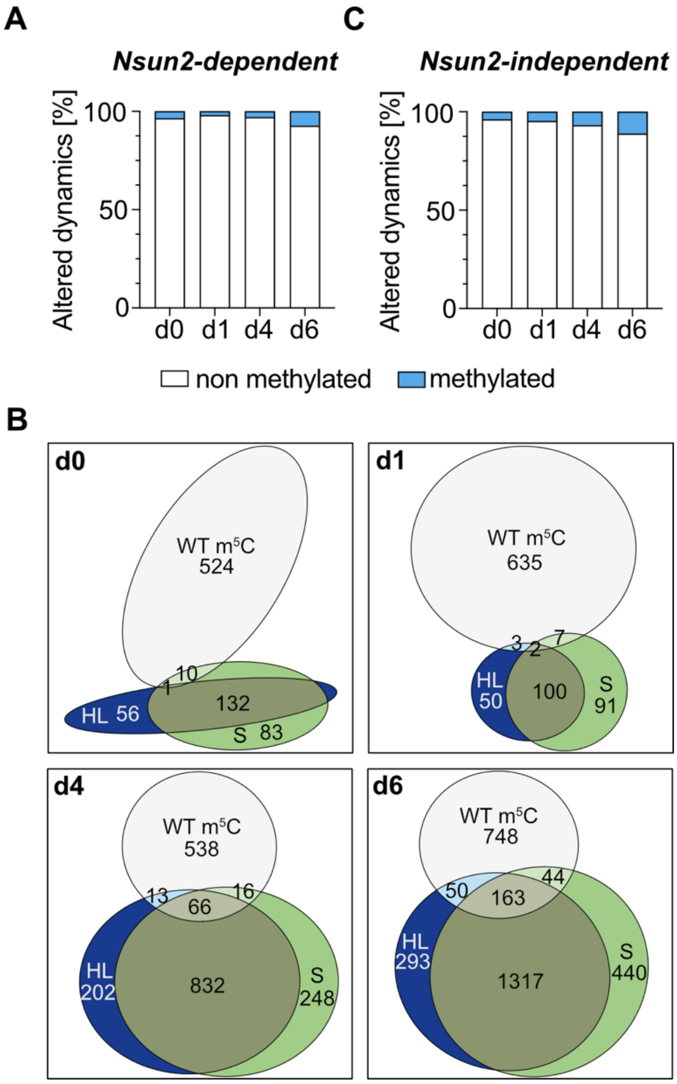

Supplementary Figure S6. Limited overlap between m^{5}C-containing transcripts and transcripts with altered turnover dynamics in *Nsun2*$^{-/-}$ compared to *WT*. (**A**) Proportion of transcripts with altered HL and S in *Nsun2*$^{-/-}$ compared to *WT* cells containing Nsun2-dependent methylation. (**B**) VENN diagrams depicting the relationship between m^{5}C-containing transcripts in *WT* (Nsun2-dependent and independent) and transcripts with significantly altered HL and/or S in *Nsun2*$^{-/-}$ compared to *WT* cells at the indicated times of differentiation. HL/S values with absolute change |log2fc| > 1 and absolute region of practical equivalence |ROPE| > 0.45 were considered. (**C**) Proportion of transcripts exhibiting Nsun2-independent methylation with altered HL and S in *Nsun2*$^{-/-}$ compared to *WT* cells.

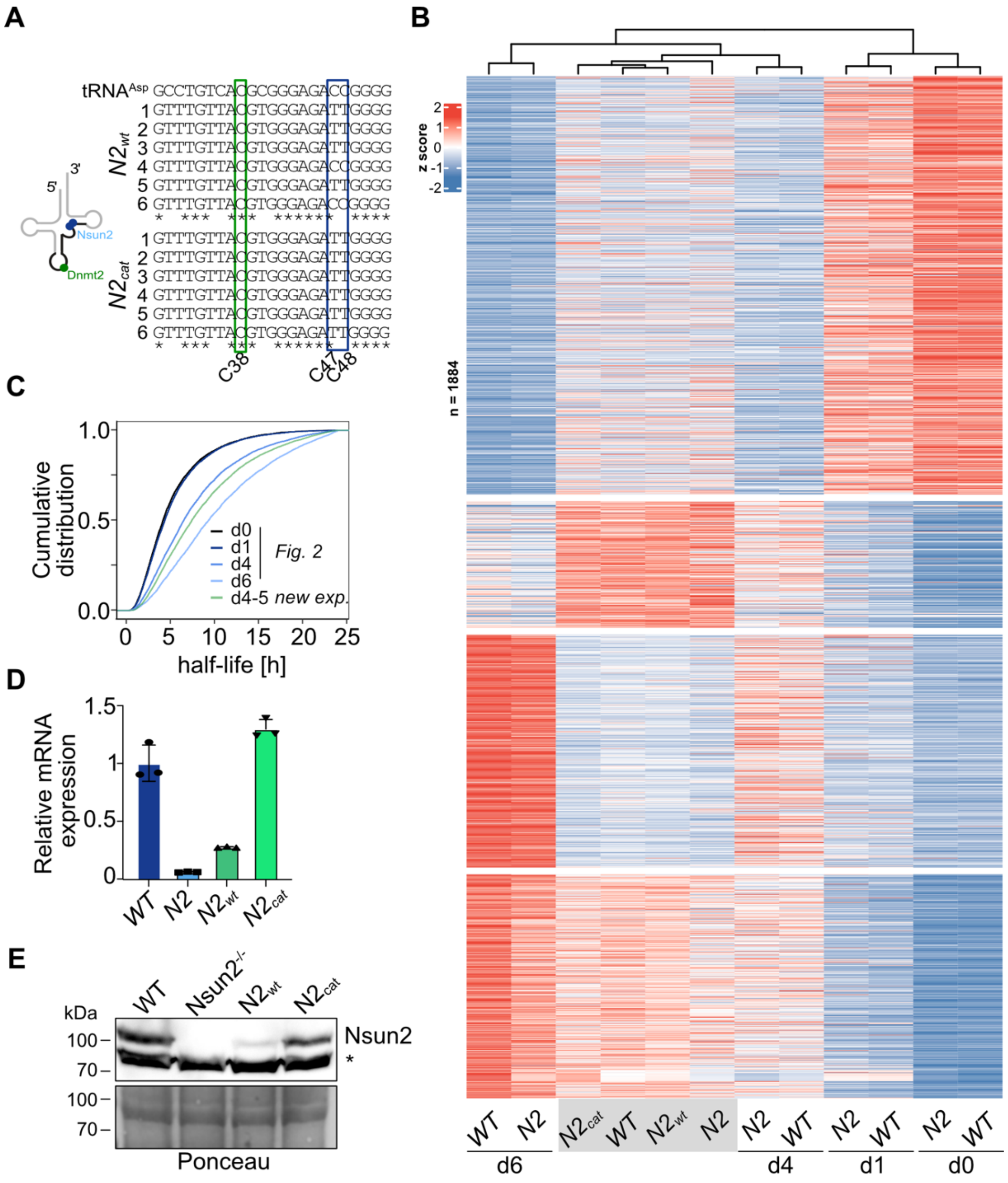

Supplementary Figure S7. NEC differentiation experiment with ESC lines expressing catalytically active or inactive Nsun2 in an *Nsun2*$^{-/-}$ background. (**A**) PCR-mediated BS-seq of tRNA^{Asp} in *N2*$_{wt}$ and *N2*$_{cat}$ rescue ESCs. *N2*$_{wt}$ cells showed partial rescue of m5C at C47, C48, while no methylation of these positions was detected in *N2*$_{cat}$ cells. Blue box, Nsun2 target sites C47, C48; green box, Dnmt2 target site C38. (**B**) Heatmap of expression profiles during NEC differentiation. Samples from the TUC-seq experiment shown in Figures 1 and 2 (d0-d6) and samples from a separate experiment involving *Nsun2*$^{-/-}$ cell lines expressing N2$_{wt}$ or N2$_{cat}$ (grey box) were combined. Transcripts displaying significant expression changes during differentiation (|log2fc| > 1, $p < 0.05$; DESeq2 likelihood ratio test) were subjected to clustering analysis. The results suggest that the differentiation stage of the rescue samples is between d4 and d6 of the original experiment. (**C**) The cumulative distribution of transcript half-lives places the experiment involving the rescued cell lines between d4 and d6 of NEC differentiation of the original experiment. *WT* profiles of both experiments are shown. (**D**) qPCR analysis of mRNA expression levels of the *N2*$_{wt}$ and *N2*$_{cat}$ transcripts in the *Nsun2*$^{-/-}$ (*N2*) background. $2^{-\Delta\Delta Ct}$ values were calculated with *TATA binding protein* (*TBP*) as the reference gene. Mean ±SD of three technical replicates is shown. (**E**) Western blot analysis of Nsun2 expression in the indicated mESCs. A section of the Ponceau-stained membrane is shown to confirm equal loading. Asterisk denotes an unspecific signal.

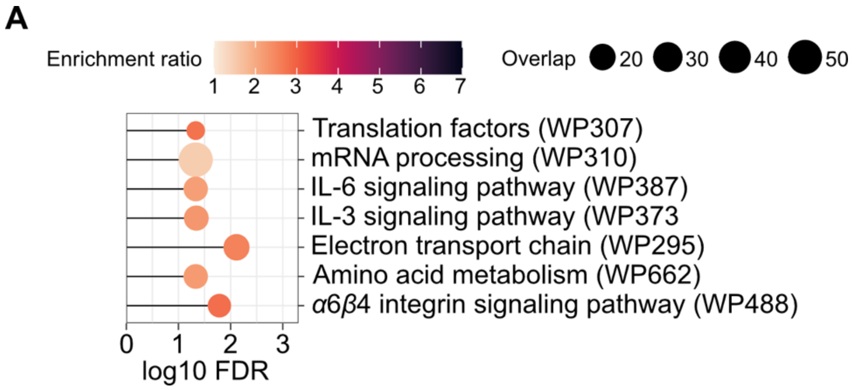

Supplementary Figure S8. Loss of Nsun2 causes dysregulation of translation. (**A**) Wikipathway overrepresentation analysis of transcripts with increased stability (log2fc>1, ROPE>0.45) in *Nsun2*$^{-/-}$ cells at d4 of NEC differentiation compared to d0, but unchanged stability in *WT* cells. Colors denote the enrichment ratio, and circle size corresponds to the number of overlapping genes. Wikipathway identifiers are shown in parentheses.
